# Supplementary material for: A gravity-based three-dimensional compass in the mouse brain
Source: Nat Commun. 2020 Apr 15;11:1855. doi: 10.1038/s41467-020-15566-5 (PMC7160108; doi:10.1038/s41467-020-15566-5)
Supplement: Supplementary file 1 — Supplementary Information [file 41467_2020_15566_MOESM1_ESM.pdf]

SUPPLEMENTARY INFORMATION

A gravity-based three-dimensional compass in the mouse brain

Dora E Angelaki[1,2], Julia Ng[2], Amada M Abrego[2], Henry X Cham[2], Eftihia K Asprodini[3], J David Dickman[2,4] and Jean Laurens[2]

[1] Center for Neural Science and Tandon School of Engineering, New York University, NY, USA.

[2] Department of Neuroscience, Baylor college of Medicine, Houston, Texas, USA.

[3] Department of Pharmacology, Faculty of Medicine, School of Health Sciences, University of Thessaly, Larissa, Greece

[4] Department of Electrical and Computer Engineering, Rice University, Houston, Texas, USA.

Address for correspondence:

Dr. Dora E. Angelaki

Email: da93@nyu.edu

Center for Neural Science, Meyer 901

New York University, NY 10003

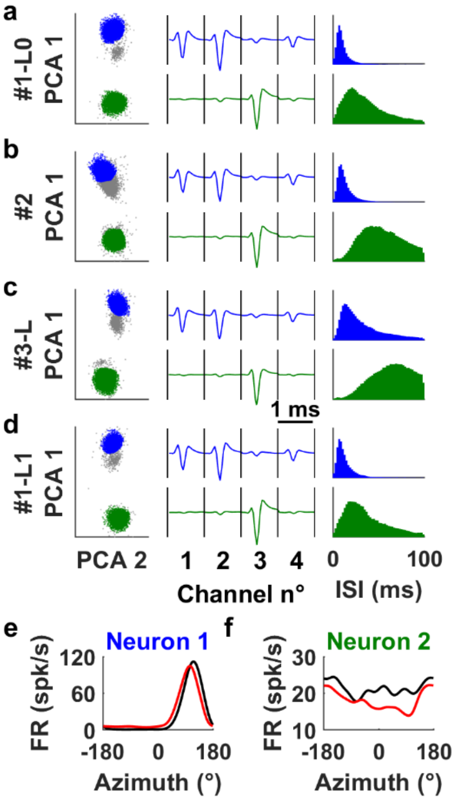

Supplementary Figure 1: Recording stability across sessions.

(a-d) Two neurons (color-coded in blue and green; animal H68M, CIN) were recorded simultaneously during Experiment 1-L0 (a), Experiment 2 (b), Experiment 3-L (c) and Experiment 1-L1 (d). Left panels: spikes were extracted by manual clustering based on the maximum (peak) and minimum (valley) voltage of the spikes, combined with factor analysis (the first two components, PCA 1 and PCA 2, are shown here). When represented on a 2D plot, the spikes form two clearly distinct clusters (blue and green). Grey dots represent unsorted events that result from noise and background activity. Middle panels: average spike waveforms across all channels. Right panels: the Inter-spike interval (ISI) histograms are conserved across Experiment 1-L0 and 1-L1 (a, d) but shift rightward during Experiment 2 (for neuron 2) and Experiment 3-L for both neurons. This shift reflects the reduction of average firing rate during Experiments 2 and 3 due to the general attenuation of neuronal firing in the rotator (see **Supplementary Fig. 8a,b**).

(e-f) Azimuth tuning curves of the neurons, recorded in the initial (black) and second (red) freely moving session, i.e. before and after Experiments 2 and 3-L, are similar. This comparison serves as confirmation of recoding stability across sessions (see also **Supplementary Fig. 3**).

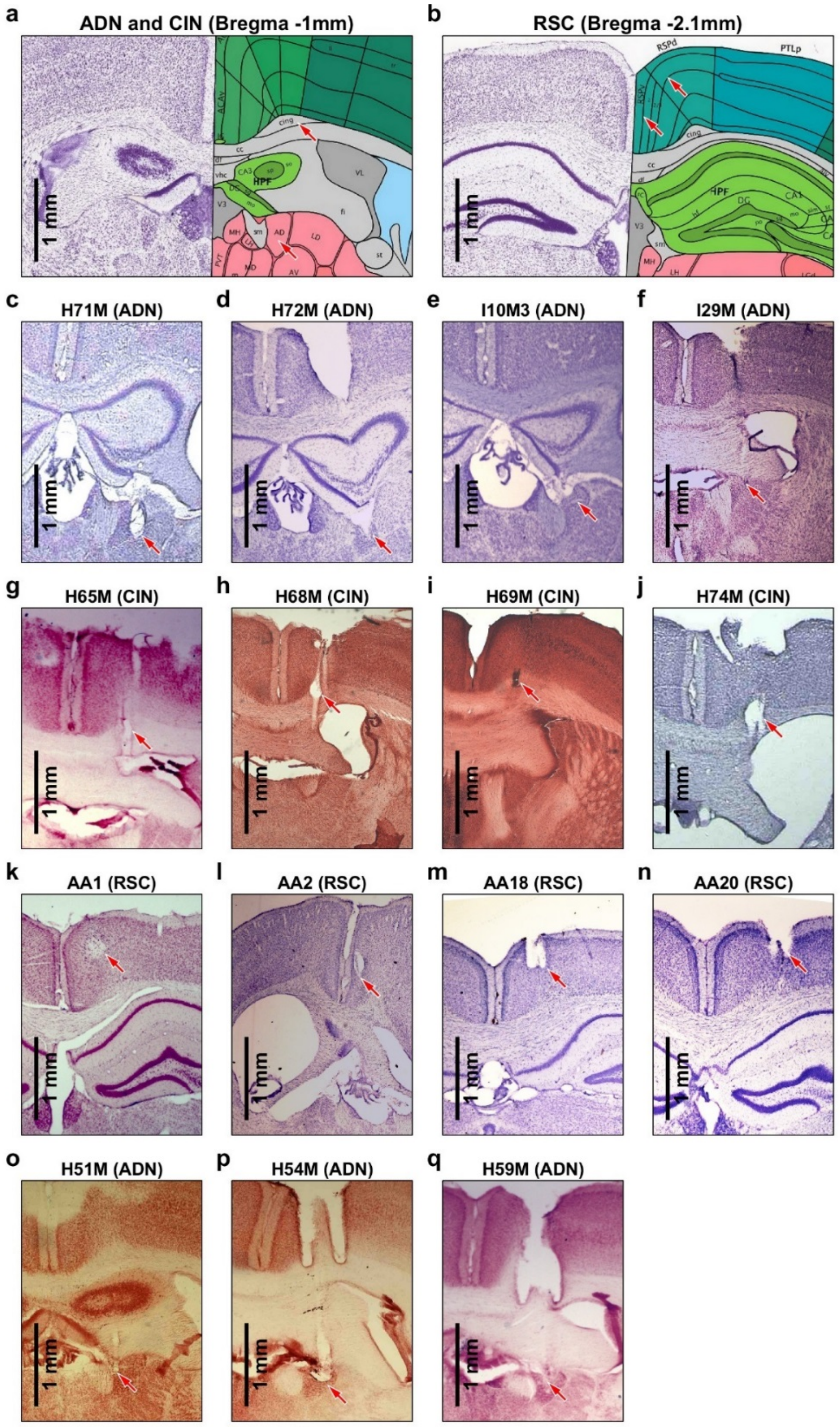

Supplementary Figure 2: Histological localization of neuronal recordings.

(a,b) Annotated histology slides (image credit: Allen Institute[1]; available from http://atlas.brain-map.org/atlas?atlas=1&plate=100960268 and http://atlas.brain-map.org/atlas?atlas=1&plate=100960224). The location of the sections relative to Bregma are

indicated in the title. Red arrows indicate the ADN (labelled "AD"), CIN (labelled "cing") in (a), the I-IIth layer of the granular RSC (labelled RSCv; left arrow) and the Vth layer of the dysgranular RSC (RSCd; right arrow) in (b). Note that the ADN extends from Bregma -0.4mm to Bregma -1.1mm. Nissl-stained sections of all animals included in this study are shown; tetrode tracks are indicated by arrows.

(c-f) Recordings in the ADN. In all animals, the ADN appears as a characteristic triangular-shaped and densely stained nucleus. In H71M, H72M and I10M3, the ADN appears below the hippocampus, i.e. more caudal than usually indicated in brain atlases[1,2]. However, we confirmed that the nucleus marked by an arrow is indeed the ADN in each mouse by examining all microscopic sections and locating the anterior extremity of the thalamus as well as the anterior and posterior extent of the ADN.

(g-j) Recordings in the cingulum fiber bundle.

(k-n) Recordings in the RSC (AA1; AA18; AA20: dysgranular RSC; AA2: granular RSC; layer indeterminate).

(o-q) Additional recordings in the ADN. These animals were used in preliminary experiments where the rotator contained limited visual cues, precluding the measurement of azimuth tuning in the rotator. These animals are included only in **Supplementary Fig. 18**.

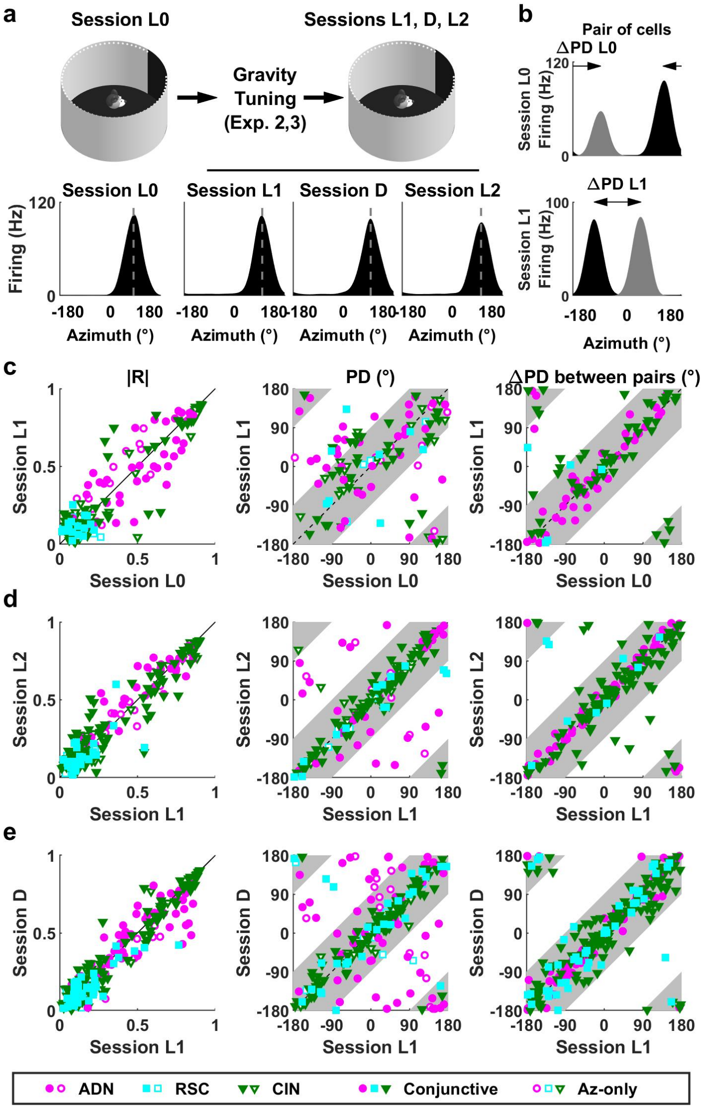

Supplementary Figure 3: Response of Azimuth-tuned cells during unrestrained motion. As summarized in this analysis, azimuth-tuned cells conform to well-established properties[3,4].

(a) Illustration of the sequence of recordings and example cell. At the beginning of an experimental day, azimuth tuning is recorded in light as the mouse forages freely in a circular arena (session L0). The mouse is then transferred to the platform and rotator to characterize its 3D tuning (Exp. 2-3; see **Supplementary Table 2** and **Methods**), upon completion it is returned to the freely moving arena and azimuth tuning is measured in light again (session L1), then in darkness (D), then again in light (L2). An example azimuth-tuned cell with stable preferred direction (PD) in all sessions in the arena (L0, L1, D, L2).

(b) Two simultaneously recoded cells (grey and black tuning curves) that changed PDs between sessions L0 and L1 (see also other mouse studies[5]). Importantly, both cells shift together, such that the difference angle between their PD (ΔPD) remains constant. Thus, comparing ΔPD across cell pairs allows testing whether azimuth-tuned cells form a coherent neuronal compass even when this compass drifts from one session to another.

(c) Azimuth response stability between sessions L0 and L1. Left: There is no significant difference in tuning strength (Mean vector length |R|: signed rank tests, p>0.5 for all groups; Bonferroni correction applied; data from all cells significantly tuned to azimuth in at least one session; n = 54 ADN; 33 RSC; 83 CIN). Middle: Comparison between the PD of individual cells. Only HD cells significantly tuned (p<0.01) in both sessions are included. PDs of a small subpopulation may drift between session L0 and L1 (PD shift > 90° in 14/46 ADN; 3/12 RSC; 7/56 CIN cells, i.e. 21% cells total). Grey bands represent sectors where the PDs shift by less than 90°. Right: ΔPD between pairs of simultaneously recorded cells. Only cells significantly tuned (p<0.01) in both sessions are included. PD differences are stable (<90° shift) in 93% (45/46 ADN; 4/5 RSC; 50/55 CIN) of cells pairs. Thus, although PD may shift between L0 and L1, the PD of all cells tend to shift together, in line with predictions of an attractor network[4].

(d) Azimuth response stability between sessions L1 and L2 (same legend as in c). There is no significant difference in tuning strength (p=0.08 for ADN, p>0.5 for other groups; n=55 ADN; 39 RSC; 135 CIN). Only 11% of cells (15/48 ADN; 2/19 RSC; 1/92 CIN) drift more than 90°. PD differences are stable (<90° shift) in 96% (61/61 ADN; 7/9 RSC; 102/108 CIN) of cell pairs. Thus, PD are more stable between L1 and L2 compared to L0 and L1, likely because of the shorter time interval between L1 and L2 and/or the use of 3D stimuli in-between sessions L0 and L1.

(e) Azimuth response stability between sessions L1 and D (same legend as in c,d). Tuning strength is slightly attenuated in darkness in RSC (linear regression slope=0.74, $p < 10^{-4}$; n=81) but not in other areas (ADN: n=70, p = 0.14; CIN: n=143; p = 0.3). Only 13% (25/63 ADN; 4/44 RSC; 0/113 CIN) of PDs drift more than 90°. PD differences are stable (<90° shift) in 99% (84/84 ADN; 45/46 RSC;146/148 CIN) of cell pairs.

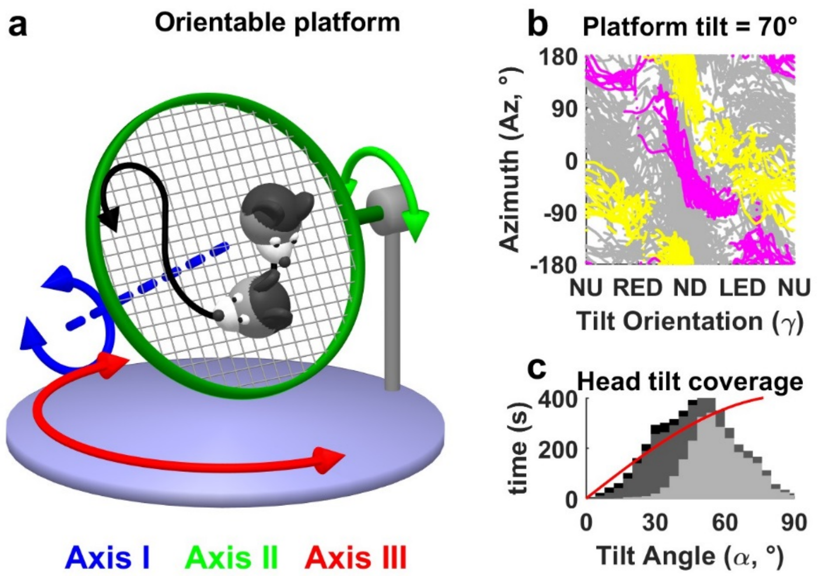

Supplementary Figure 4: Freely moving protocol for measuring 3D tuning in unrestrained, mice.

(a) Illustration of the 3D orientable platform setup: mice walk freely (black arrow) on a meshed platform that can rotate around 3 axes (blue, green, red). Walking on the platform changes tilt orientation (γ; see **Supplementary Fig. 5**) and azimuth simultaneously. Rotating the base (Axis III) changes azimuth but not tilt orientation. Axis II is used to change tilt angle (α; see **Supplementary Fig. 5**). Recordings are performed in 5-8-minute blocks where mice walk freely while axis II-III are set to a static position. Axis I is repositioned within each block, as explained below.

(b) Distribution of azimuth (Az) and tilt orientation (angle γ) in eight 5-min blocks where the platform was tilted 70° (average head tilt = 60±15°, as mice tend to partially compensate with their head). Yellow, magenta: data recorded during two 5-min blocks corresponding to different configurations (same tilt angle, but different positions of Axis III). Azimuth and tilt orientation vary together as animals walk within one block, but azimuth is offset when Axis III is rotated between blocks, thus allowing to scan the entire tilt orientation/azimuth plane.

(c) Distribution of head tilt angle (α) in the same recording session (black/grey/light grey: data collected with the mesh tilted 0°, 45°, 70°, respectively). Red curve: distribution required for uniform sampling of tilt orientation, illustrating relatively uniform sampling up to ~60°). Note that, if only Axis III was rotated to change azimuth between blocks, the same physical portions of the platform would always be oriented downward (or upward, or horizontally). In this situation,

local orientation on the platform itself would correlate with tilt relative to vertical. This could create a confounding factor since azimuth-tuned cells may potentially be referenced to the platform itself instead of distal cues[6]. To eliminate this confound, we rotated Axis I randomly in the middle of each block. Because of this added complexity, earth-horizontal azimuth tuning in **Fig. 2a** was evaluated based on data recorded in the arena.

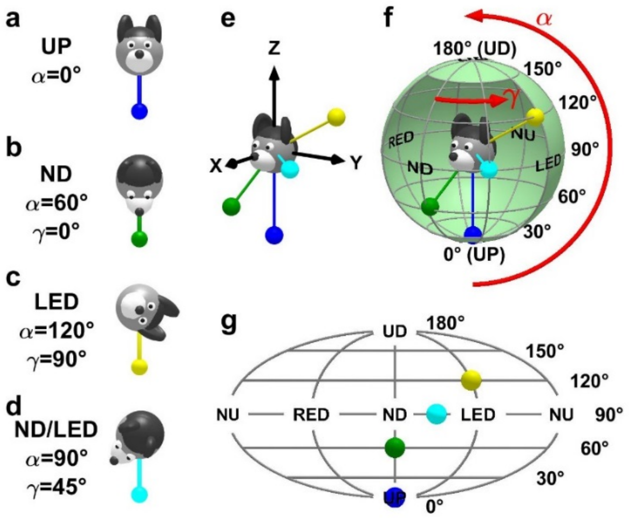

Supplementary Figure 5: Coordinate systems for tilt relative to vertical. A convenient way to express head orientation to vertical is to represent the orientation of an allocentric vertical vector in egocentric coordinates. In this study, we chose a downward-pointing vertical vector as a reference. We generally refer to this vector as the gravity vector **G** for simplicity, although the approach can be applied to any other vertical reference. This vector is encoded in 3D Cartesian coordinates for data analysis and modeling purposes. However, since its length is constant, it is restricted to a 2D sphere surrounding the head. Therefore, we use a simpler spherical coordinate system to describe head tilt and represent tilt tuning curves.

(a-d) Four example tilt orientations, expressed in a spherical coordinate system (α, γ) where α is the tilt angle and γ is tilt orientation: γ = 0° and γ = 180° correspond to nose-down (ND) and nose-up (NU) tilt; γ = 90° and γ = -90° correspond to left-ear-down (LED) and right-ear-down (RED) tilt. The colored pendulum/ball represents the gravity vector.

(e) Representation of the gravity vector in (a-d) in egocentric Cartesian coordinates.

(f) Spherical topology of tilt orientation. When head tilt spans all possible orientations, the tip of the gravity vector spans a sphere surrounding the head. The tilt variable α corresponds to the latitude on the sphere. Upright (UP, α=0°) and upside-down (UD, α=180°) orientations correspond to the lower and upper pole respectively. The orientation variable γ corresponds to the longitude. 90° tilt in ND, LED, NU and RED orientations are marked.

(g) Planar representation of the sphere using an equal-area Mollweide projection. The 4 tilt orientations in (a-d) are marked with color balls.

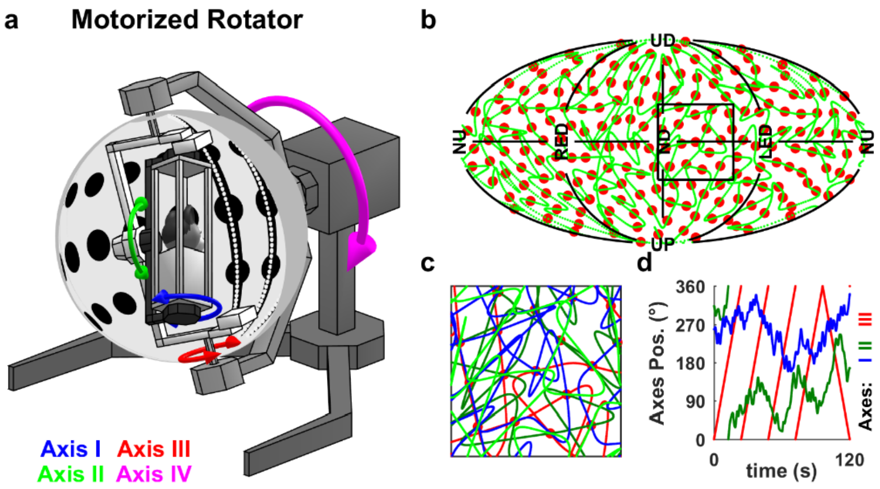

Supplementary Figure 6: Rotator and protocol for measuring 3D tuning in restrained mice.

(a) Illustration of the motorized rotator; the 4-rotation axes are indicated by colored arrows.

(b) Pseudo-random trajectory (green curve) used to measure tilt tuning. The trajectory visits 200 uniformly distributed tilt positions (red dots). The full protocol scans the entire tilt space 8 times by running through 4 distinct trajectories, each of which is ran twice in opposite directions.

(c) Detail of the highlighted square in (b), with 4 distinct trajectories.

(d) Position of the rotator's 3 inner axes during a 2 min segment of the motion. Axes I (inner yaw, blue) and II (middle, pitch/roll, green) are used to manipulate 2D head tilt relative to vertical, while axis III (outer yaw, red) is used to continuously vary azimuth. Axis IV is used to tilt the setup in Experiment 3-T (**Supplementary Table 1**).

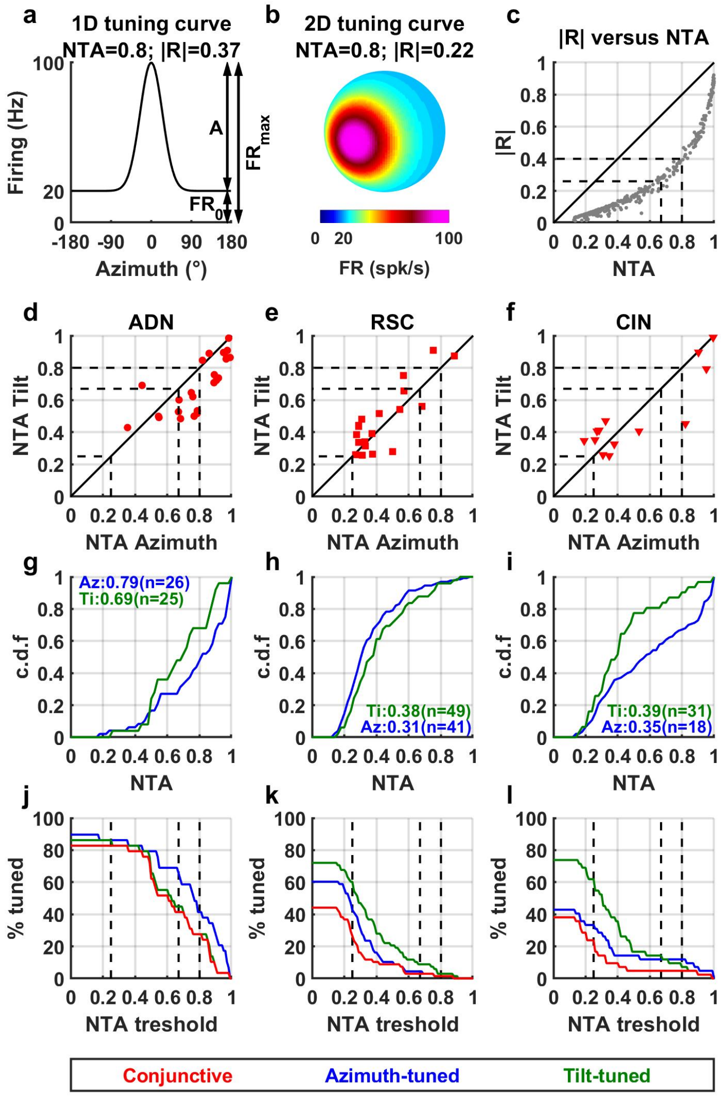

Supplementary Figure 7: Tuning strength in tilt- and azimuth-tuned cells.

(a) We define a measure of tuning strength called normalized tuning amplitude (NTA), illustrated here for a 1D tuning curve. We decompose firing rate into a baseline firing FR_0 and a modulation amplitude A. The peak firing rate is $FR_{max}=FR_0+A$. Normalized tuning amplitude is defined as $NTA=A/FR_{max}$. The normalized tuning amplitude of this example is NTA = 0.8; and the mean vector length is |R|=0.37.

(b) Illustration of a 2D tuning curve on a sphere, used to model tilt tuning. This distribution has identical baseline, amplitude and standard deviation as in (a) and therefore, the normalized tuning amplitude is also NTA = 0.8. However, the mean vector length is |R|=0.22; i.e. lower than in (a). This is because the area of baseline firing on a sphere (cyan in b) amounts to a larger portion

of the tuning curve than in a 1D tuning curve, resulting in a lower |R| value. Therefore, the mean vector length is inappropriate for comparing azimuth (1D) and tilt (2D) tuning. Furthermore, the mean vector would be even lower in double-peaked curves, where the second peak would bring the mean vector even closer to zero. Finally, the mean vector is disproportionately influenced by a cell's baseline firing rate even in 1D, for instance |R| in panel (a) would increase from 0.37 to 0.91 if the cell's baseline firing was set to 0. These limitations motivated us to develop the NTA measure.

(c) Comparison of the mean vector length (|R|) and normalized tuning amplitude for azimuth tuning in Experiment 1-L (**Supplementary Table 2**; data from all significantly-tuned cells). Although the scaling between the two measures is non-linear, there is a clear relationship between them, indicating that they provide the same information. Therefore, the normalized tuning amplitude is as suitable as the mean vector for quantifying tuning strength, with the advantage that it allows for a fair comparison between 1D and 2D tuning curves.

(d-f) Comparison of the normalized tuning amplitude of azimuth and tilt tuning for all conjunctive cells (as in **Fig. 2a**). Previous studies[5,7] have classified neurons as HD cells when |R|>0.26 or |R|>0.4. We infer from the population response in (c) that these values correspond to NTA≈0.67 and NTA≈0.8, respectively. In the present study, we classified cells as azimuth or tilt-tuned using statistical criteria: if tuning passed a shuffling test (at p<0.01), as long as NTA>0.25 (to exclude low-modulating cells). The three thresholds (NTA=0.25, 0.67, 0.8) are indicated by vertical dashed lines. Although the statistical criterion we used was more inclusive compared to the fixed threshold of previous studies, the 3D tuning properties described here are found in those cells that pass the more restrictive criteria of previous studies, as detailed in subsequent panels.

(g-i) Cumulative distribution of NTA for all azimuth-tuned (blue) and tilt-tuned (green) cells that passed the shuffling test (not only conjunctive cells as in panels d-f). Median values of NTA and number of cells are indicated in the panels. The median values of tilt and azimuth tuning are comparable in all regions.

(j-l) Percentage of cells that would be classified as azimuth-tuned (blue), tilt-tuned (green) and conjunctive (red) by passing the shuffling test (p<0.01) and exceeding a variable NTA

threshold, expressed as a function of that threshold, but including all recorded cells in both the arena and platform setups. Note that the threshold value of NTA =0.25 used in the present study allows for a large fraction of cells to be classified as azimuth-tuned, tilt-tuned or conjunctive (e.g. 86% azimuth-tuned and 86% tilt-tuned in ADN). Using a more stringent threshold (e.g. NTA=0.8) to select cells with very vigorous responses, we find that 41% ADN cells are classified as azimuth-tuned, 28% as tilt-tuned, and 28% as conjunctive. Thus, even when a stringent criterion is used, over a fourth of ADN cells exhibit large 3D responses. A sizeable, but lower, fraction of CIN cells (12% azimuth-tuned, 7% tilt-tuned, 5% conjunctive) exhibit similar strong responses. In contrast, only a minority (<3%) of RSC cells pass this threshold, indicating that significant HD responses exist in RSC but are generally weaker than in ADN, which is a known property already (Chen et al. 1994). Importantly, the proportion of tilt-tuned cells is typically higher that Az-tuned cells in all areas, regardless of NTA threshold (green vs. blue curves).

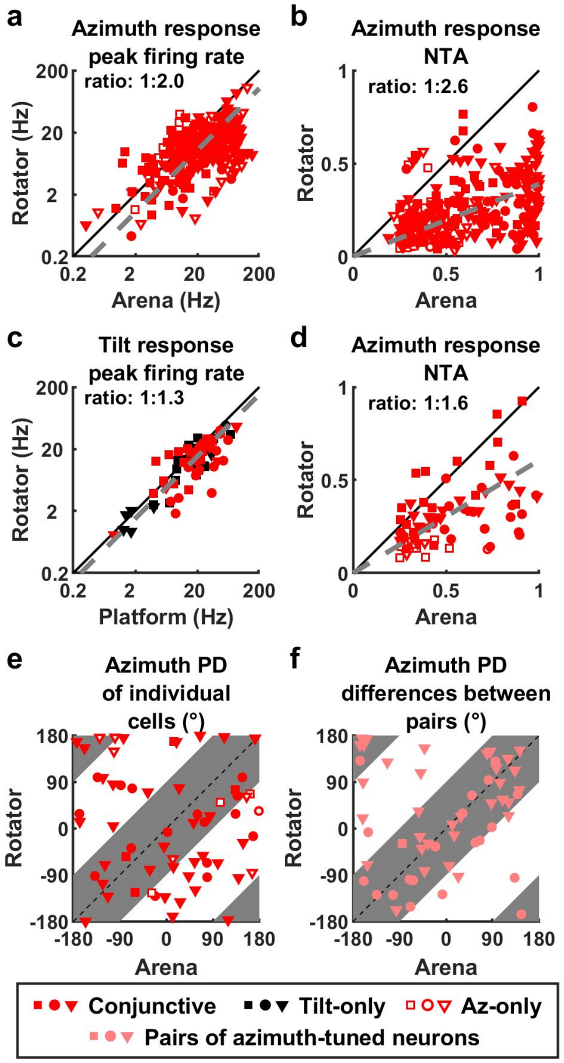

Supplementary Figure 8: Azimuth and tilt responses when moving freely and restrained.

(a,b) Comparison of azimuth response amplitude when moving freely in the arena (Experiment 1-L0,1,2; **Supplementary Table 2**) and when restrained in the rotator (Experiment 3-L, considering data for tilt < 45°). Data from cells significantly modulated to azimuth in Experiment 1 and recorded during Experiment 3-L (n=303). Responses were attenuated in two ways when mice were restrained: the peak firing of the cells was reduced (a; median amplitude ratio= 1:2 [1:7.3 - 1:2.2] CI; grey) and the NTA (peak to trough amplitude divided by peak firing) was also reduced (b: median amplitude ratio= 1:2.6 [1:2.4 - 1:2.8] CI; grey).

(c-d) Comparison of tilt response amplitude when moving freely on the platform (Experiment 2; **Supplementary Table 2**) and when restrained in the rotator (Experiment 3-L; re-analyzed based on data when head tilt < 60° to match the range of tilt sampled in Experiment 2).

Data from cells significantly modulated to tilt when moving freely and recorded during Experiment 3-L (n = 70). Both the peak responses and the NTA were attenuated (peak: median amplitude ratio= 1:1.3 [1:1.2 1:1.5] CI; NTA: median amplitude ratio= 1:1.6 [1:1.5 1:2] CI). Together, panels (a-d) indicate that restraining mice leads to an attenuation of both azimuth and tilt responses, both in terms of peak firing and in terms of response modulation relative to firing (i.e. NTA). Azimuth tuning is attenuated to a larger extent than tilt tuning (Wilcoxon rank sum tests, peak response: $p=0.002$; NRA: $p<10^{-8}$), which is the reason tilt tuning curves can be reliably measured in the rotator in most neurons, but azimuth tuning curves only in a minority of neurons (**Fig. 2c**).

(e,f) Inconsistency in azimuth PDs, but consistency of difference in azimuth PD between pairs of cells across setups. The arena and the rotator are different setups located in separate rooms and don't share a common azimuth reference. Therefore, the azimuth compass may anchor to a priori random orientations in each setup, which would cause the PD of individual cells to vary randomly. Accordingly, PDs shift by more than 90° in 29/63 cells (panel c, $p=0.6$, Binomial test). However, if azimuth-tuned cells are part of a neural compass, then the PD of simultaneously recorded cells should remain anchored one relative to the other. Therefore, the difference in PD between pair of cells should be identical in the arena and in the rotator. Indeed, PD differences in the arena vs. rotator were significantly conserved in panel d (within 90° in 44/54 pairs of cells, $p<10^{-5}$, Binomial test). Note, however, that unlike the azimuth compass that anchors to visual landmarks, a tilt compass anchored to gravity (**Fig. 1a**), should have identical PDs on the platform and in the rotator. This is indeed the case, as shown in the pixel-by-pixel correlation of the fitted tilt tuning curves up to 60° tilt that could be tested in both setups (**Fig. 2d**; We used the correlation analysis for tilt tuning, because the PD of most cells can't be measured on the platform, which is restricted to 60° tilt. Also note that together, panels a-d and **Fig. 2** indicate that the spatial tuning characteristics of both azimuth and tilt tuning are conserved across free locomotion and restrained, passive motion, and that, other than the smaller response magnitude, the 3D responses measured in the rotator are representative of the neurons' natural responses.

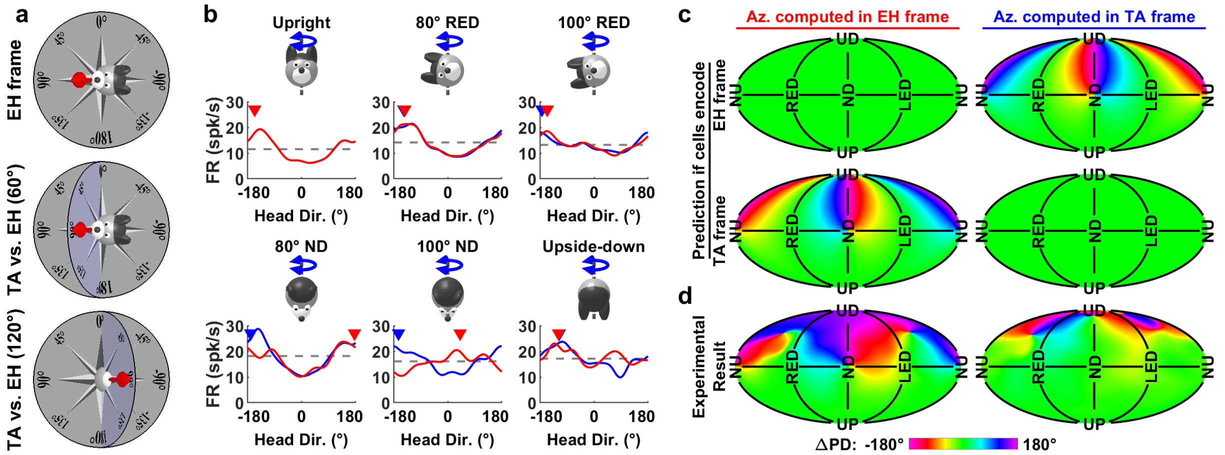

Supplementary Figure 9: Azimuth tuning is spatially invariant when expressed in a TA frame.

(a) Illustration of the EH and TA model (equivalent to the dual-axis rule[7,8]). In an EH frame (top panel), head direction is projected onto the EH plane (grey). In a TA frame, head direction is measured in a compass (blue) that is coplanar with the head horizontal plane and oriented such that the azimuth measured in the EH and TA frame coincide along the line of intersection of both planes (the 0-180° axis here). In other words, the TA frame is anchored to the allocentric reference frame along the earth-horizontal direction. It is defined by rotating a horizontal compass to align with head direction, instead of projecting head direction onto the horizontal plane (EH frame). In the example orientations shown here, the head pitches upward by 60° (middle panel) and 120° (bottom panel). In a TA frame, it faces 90° in both panels. When projected onto the EH plane, its direction reverses from 90° to -90° when pitch angle exceeds 90°, as reported by Finkelstein et al.[9]. Note that, if the head is facing the 0° (or 180°) direction, it would be rolling instead of pitching, and azimuth reversal would not occur since these directions coincide in the EH and TA frames. As a general rule, TA is reversed relative to EH azimuth when tilt angle exceeds 90° in the pitch plane, but not in the roll plane. In intermediate tilt planes, the difference between EH and TA azimuth depends of tilt angle (see panel c).

(b) Azimuth tuning curves of an example cell, extracted from the full 3D tuning curve measured in Experiment 3-L and computed in EH (red) or TA (blue) reference frames. Each curve represents the firing rate for all possible azimuths at a single tilt angle, which correspond to the positions attained by tilting the head to a given orientation and rotating around an earth-vertical axis (see **Supplementary Movie 1**). Note that the azimuth response of the example cell is modest

since azimuth tuning is reduced when measured in a rotator (see **Supplementary Fig. 8a,b**). In an EH frame, the cell's PD (-157°) was conserved for tilt orientations in the roll plane (-143° and -156° at 80° and 100° RED) but reversed abruptly for tilt angles larger than 90° in the pitch plane (from 177° to 41° at 80° and 100° ND). However, there is no such abrupt reversal when azimuth is computed in the TA frame.

(c) Predicted change of the cell's azimuth PD in tilted orientations (ΔPD, expressed relative to its PD when upright), displayed as a color map. For a given 3D head orientation, azimuth differs when computed in a EH or TA frame. The difference between both azimuths depends on the head's orientation relative to gravity (see **Online Methods**). If cells encode azimuth in an EH frame, we expect their PD to be invariant across all head tilts (i.e. $\Delta PD \approx 0$) when azimuth is expressed in the EH frame (upper left panel) but to vary with head tilt, and in particular to reverse when the head is pitched beyond 90° (i.e. between NU/ND and UD) when azimuth is expressed in TA frame (upper right panel). Reciprocally, if cells encode azimuth in a TA frame, we expect their PD to be invariant when expressed in a TA frame (lower right panel) but to vary when expressed in EH frame (lower left).

(d) Average ΔPD across all azimuth-tuned cells significantly tuned to azimuth in Experiment 3-L (17 ADN, 7 RSC, 39 CIN). The azimuth PD varies when expressed in an EH frame but remains invariant (except close to UD) when expressed in a TA frame, in line with predictions of the TA model. Note that the PD becomes more variable close to UD, likely because azimuth tuning amplitude is near zero close to UD (see **Fig. 3g**), making data unreliable at this orientation.

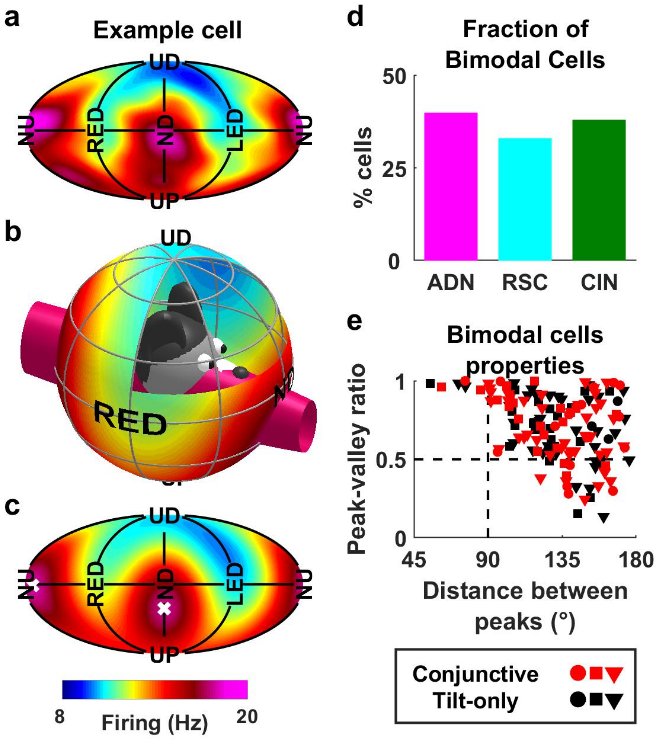

Supplementary Figure 10: The model of 3D gravity tuning explains bimodal tilt tuning curves.

(a) Example cell exhibiting two response peaks, in ND and NU orientation.

(b) 3D Gaussian tuning model. Gravity tuning is modeled as a 3D Gaussian tuning curve in egocentric Cartesian space (ellipsoid in **Fig. 4a**). In this cell, the 3D Gaussian, shown as a purple ellipsoid (indicating points located within 0.4 standard deviation from the Gaussian's center; the ellipse's color corresponds to the cell's firing rate at these points) is markedly elongated (the extremities of the ellipsoid are truncated to fit in the figure). Gravity on earth has a constant magnitude and is restricted to a 2D sphere around the head. The ellipsoid intersects this sphere at two positions (close to ND and NU orientation, i.e. when the gravity vector is aligned with the X axis).

(c) Same tuning curve as in (b), where the sphere has been projected onto the figure's plane. The two peaks are marked by white dots. The tuning curve matches the raw tuning curve in (a) (correlation coefficient ρ=0.98). Thus, from a practical point of view, the cell appears bimodal as it responds preferentially at two distinct head tilts; but from a mechanistic point of view its tuning can be explained by processing sensory signals through a unimodal Gaussian distribution.

(d) We identified bimodal tuning by fitting all cells with the 3D Gaussian model and counting the number of local maxima on the 2D tilt tuning curve (i.e. on the sphere). All cells have either one (unimodal) or two (bimodal) local maxima. The proportion of bimodal cells in each region is shown. Overall, 36% (141/388) tilt-tuned cells were bimodal. This proportion is similar in each region (Chi square test, p=0.56, χ^2=1.15, 2 dof), across conjunctive and tilt-only cells (Chi square test, p=0.68, χ^2=0.17, 1 dof), and across cells tuned in the pitch or roll plane (Chi square test, p=0.29, χ^2=1.14, 1 dof).

(e) We further characterized bimodal tuning curves by computing the angular distance between the two peaks (abscissa) and the ratio of the peak-valley amplitude of the two peaks (smallest/largest peak; ordinate). In the example cell in (c), the distance is 151°, i.e. the two peaks are almost opposed, and the amplitude ratio is 0.97, i.e. the two peaks have nearly equal magnitude. We find that the two peaks are well separated (distance > 90°, 135/144 cells, 96%) and have comparable magnitudes (ratio > 0.5, 116/144 cells, 83%) in most bimodal cells.

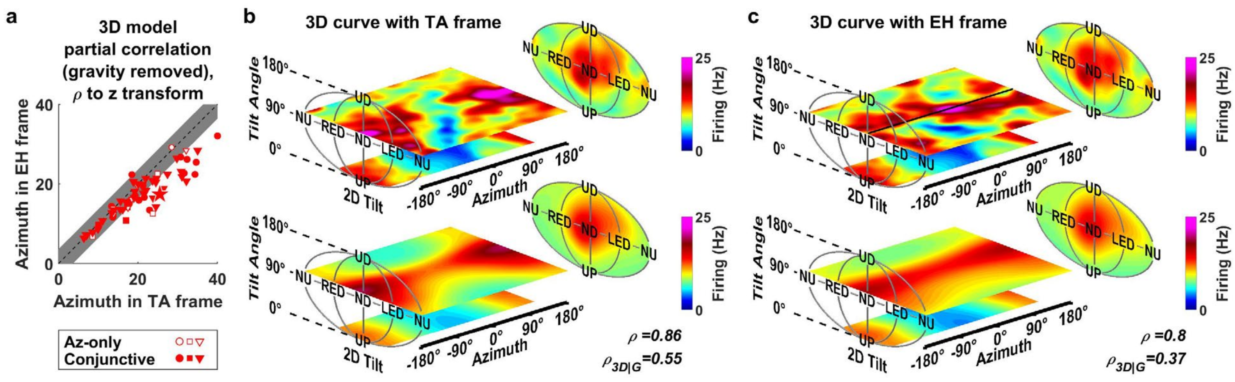

Supplementary Figure 11: Comparison of 3D model fitting with azimuth in TA or EH frame.

(a) Partial correlation of the model fits (shown as z-score), with the correlation attributable to gravity removed so that the partial correlation reflects how the model fits azimuth tuning in 3D. Data for all Az-tuned neurons that maintained their azimuth tuning in the rotator (Experiment 3-L) when the head is close to upright (<45° tilt; 17 ADN, 7 RSC, 39 CIN). Grey band: zone where partial correlations are not significantly different at p<0.01. Partial correlations were significantly higher when azimuth was expressed in a TA frame in 24/63 neurons, and significantly higher in a EH frame in only 1 ADN neuron (in this neuron, the difference between both frames was weak and vanished if only data for > 90° tilt was analyzed, indicating that it is likely a false positive). This analysis confirms that neuronal responses are more consistently expressed in a TA frame. The absence of significant difference in a large fraction of neurons (38/63) is explained by both the similarity between TA and EH frames at small tilt angles and the tendency of azimuth responses to decrease with tilt angle (see **Fig. 3f,g**). An alternative explanation, which would be that cells encode a mixture of EH and TA azimuth, may be rejected because the two frames are mutually exclusive.

(b,c) 3D tuning curve of an example neuron computed in both frames (upper panels) and corresponding model fits (lower panels). This neuron was tuned to tilt, with a PD at α=100° tilt in ND orientation (γ=-5°), as well as azimuth with a PD at -175° when upright (lower planes in the 3D curve). TA and EH frames are identical near upright and, accordingly, tuning appears similar. Next, we examine tuning at a tilt angle of 100° (upper planes), where the TA and EH frames diverge sharply (as in **Supplementary Fig. 9a,c**). In a TA frame (b), the cell still exhibited a clear azimuth tuning with a similar PD (168°) as in upright. The 3D model (lower panel) captured the 3D curve by multiplying a tilt tuning centered on 100° ND with an azimuth tuning curve centered

on 175°, leading to a total correlation of ρ=0.86 and a partial correlation of $\rho_{3D|G}$=0.55. In contrast, azimuth tuning was largely distorted when expressed in a EH frame (upper plane on panel c). In ND orientation (marked by a black line), the cell's response reversed and peaked at an azimuth of 18° (magenta). In contrast, it shifted back to ±180° on either side of the line, i.e. when head orientation neared RED and LED. This pattern, where azimuth tuning reverses in ND but not RED or LED, corresponds to the reversal of TA azimuth relative to EH azimuth (**Suppl Fig. 15a,c**) and is expected if azimuth is encoded in a TA frame. Therefore, the cell's azimuth PD was not invariant relative to head tilt when expressed in an EH frame. Since this violates the assumption of the 3D model, the correlation decreased to ρ=0.8 and $\rho_{3D|G}$=0.37 (note that the correlation didn't decrease to zero since the model could still fit azimuth tuning at low tilt angles).

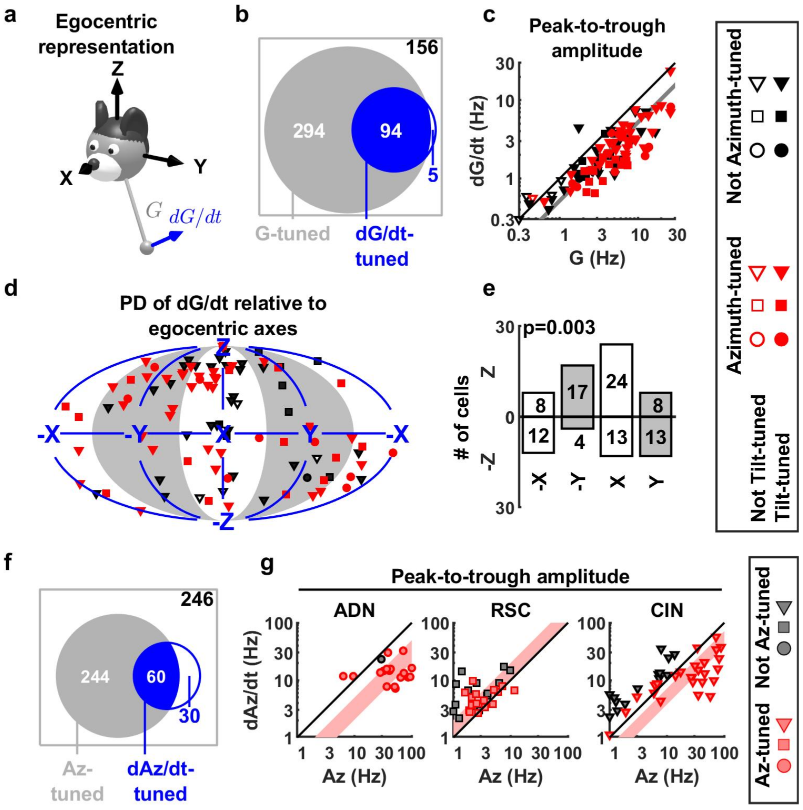

Supplementary Figure 12: Responses to tilt and azimuth velocity. We used identical criteria to assess whether cells were significantly tuned to azimuth velocity (dAz/dt) and tilt velocity (i.e. the time derivative of gravity, dG/dt) (see Methods).

(a) Gravity derivative was expressed in an egocentric (X,Y,Z) frame, similar to gravity, and the responses to gravity derivative was fitted with Gaussian functions (as in **Supplementary Fig. 10**).

(b) A small percentage of cells (99/549, 18%) exhibited significant tuning to tilt velocity (data from Experiment 3-L; **Supplementary Table 2**). Furthermore, the majority (94/99; 95%) of these were also tuned to tilt. Tilt-tuned cells were more likely to be tuned to dG/dt (Chi square test , $p<10^{-8}$, $\chi^2=34$, 1 dof).

(c) For most cells, tilt velocity responses had a lower amplitude than tilt position responses (geometrical average ratio=0.48, [0.44 0.54] CI; data from n=94 cells with significant tilt and tilt velocity tuning). There were only slight differences between areas (ratio = 0.47; 0.4 and 0.55 in ADN, RSC and CIN respectively; Kruskal-Wallis ANOVA, p=0.02).

(d) Distribution of PDs for tilt velocity. **X**, **Y**, **Z** indicate that cells fire preferentially when $dG_X/dt>0$, $dG_Y/dt>0$, and $dG_Z/dt>0$, respectively. **-X**, **-Y**, **-Z** indicate that cells fire preferentially when $dG_X/dt<0$, $dG_Y/dt<0$, and $dG_Z/dt<0$, respectively.

(e) Number of cells in all 8 quadrants of panel d. Most cells prefer $dG_Z/dt>0$, and $dG_X/dt>0$, i.e. when the gravity vector moves forward and upward in head coordinates, which corresponds from instance to ND pitch movements when starting from an upright condition. P-value based on a χ^2 test versus uniform distribution.

(f) A small percentage of cells (90/580, 16%) was also tuned to azimuth velocity (dAz/dt). 19% of azimuth-tuned cells were tuned to dAz, versus 11% of non azimuth-tuned cells (Chi square test , p=0.003, χ^2=8.6, 1 dof).

(g) For cells tuned to both Az and dAz/dt, Az velocity responses had a lower amplitude than Az position (direction) responses in ADN (median ratio: 1:3.1, [2-4.9] CI; $p < 10^{-3}$, signed rank test), but in contrast were slightly larger in RSC (median ratio 1.5:1, [1.1-2] CI; $p < 10^{-3}$). The ratio in CIN was intermediate (2:1 in favor of Az responses, [1.4-2.8] CI; $p < 10^{-5}$). The peak-to-trough amplitude of dAz/dt tuning curves, measured across the range of ±200°/s, had a median value of 13 Hz ([11-15] CI) in ADN, 5.5 Hz; [3.7-6.6] CI) in RSC. The distribution of responses in CIN resembled a mixture of ADN cells (with high Az and lower dAz responses) and RSC cells (with low Az and dAz/dt responses).

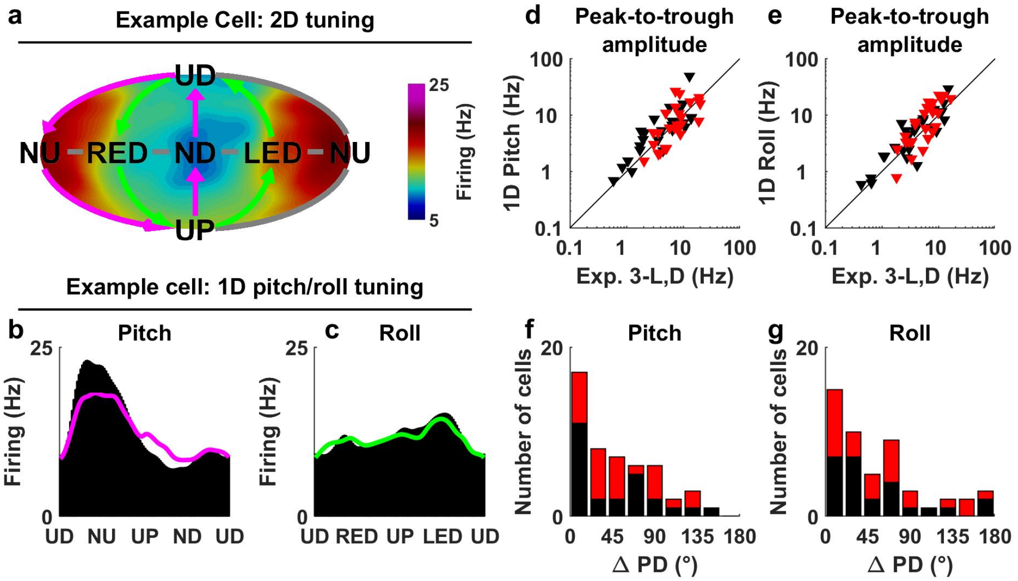

Supplementary Figure 13: Comparison between pitch/roll rotation and 3D rotation.

(a) Average tilt tuning curve of an example cell (same as in Fig. 1l). During pitch rotation, the head is tilted from upright to ND, UD, NU and back to upright, as illustrated by magenta arrows. During roll rotation, the head is tilted from upright to LED, UD, RED and back to upright (green). Rotations in the opposite sequence are also performed.

(b, c) average firing rate during pitch and roll (magenta and green curves). The firing rate measured at corresponding tilt positions during Experiment 3-L,D (3D rotations) is shown in black. Both curves match well, indicating that responses during complex 3D trajectories generalize to simple 1D rotation.

(d, e) Peak-to-trough modulation amplitude measured during pitch/roll (ordinate) vs. that predicted based on tilt tuning curves measured in Experiment 3-L,D (abscissa). Amplitudes are significantly correlated ($p<10^{-10}$ for both pitch and roll; n=50 tilt-tuned cells, data averaged across recordings in light and darkness). The responses are slightly higher during single axis rotation in roll (median=5.9 vs 3.8 Hz; $p = 10^{-3}$, signed rank test) but not pitch (median=6.4 vs 4.4 Hz; $p = 0.3$, signed rank test).

(f,g) Distributions of absolute difference in tilt preferred direction (PD) between 1D and 3D stimuli for pitch and roll planes, respectively. Both are significantly aligned with 0 (Kolmogorov-Smirnov tests to test the difference with a uniform; $p < 10^{-5}$ in both). Red symbols/bars: Azimuth-tuned cells; Black symbols/bars: Not-azimuth-tuned cells.

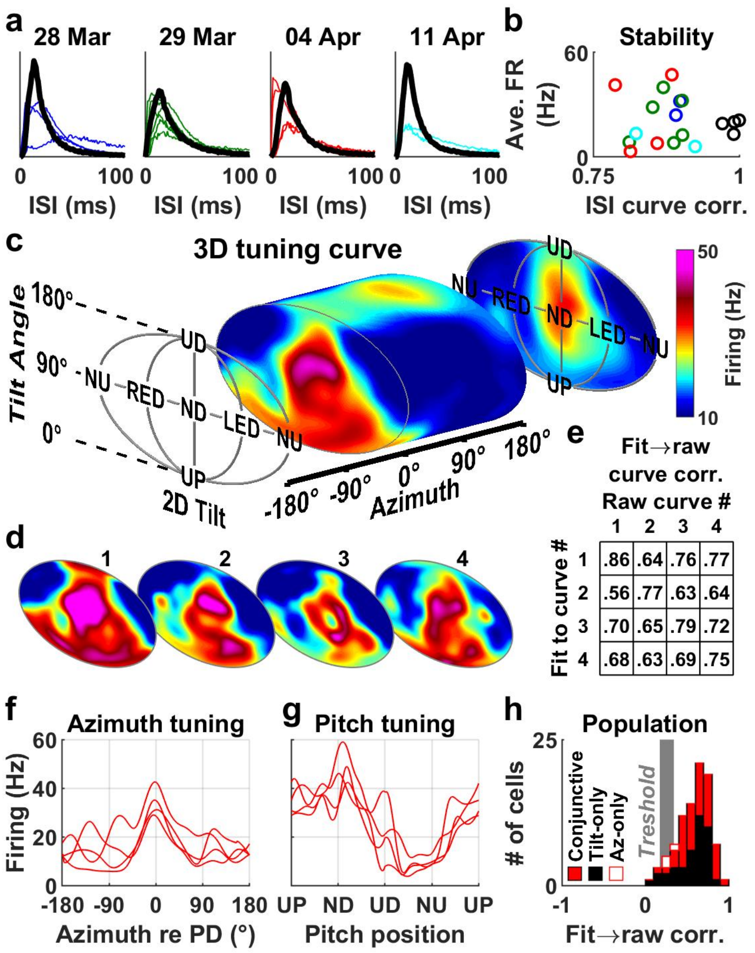

Supplementary Figure 14: Reproducibility of 3D tuning across days.

(a) ISI distribution of a neuron recorded 4 times during Experiment 3-L, on 4 distinct days spanning a 2-week period (black). The tetrode was not moved in this period. The ISI distribution of all other neurons recorded on the same tetrodes are shown in color.

(b) Identification of the neuron across the 4 recordings. We display the average firing of all neurons in (a), colored using the same code as in (a), versus the correlation between all ISI curves and the black ISI curve on March 28. The black dots form a cluster on the right side of the graph, indicating that the shape of the ISI curve and the cell's firing rate are distinct enough to identify across days. Note that we used only spike waveforms, mean firing and ISI distribution to identify neurons across multiple experiments within a single day. In contrast, we also considered tuning curves when identifying neurons across separate days, and only considered spiking activity recorded in separate days to originate from the same neurons if 3D tuning curves were similar. Thus, we were able to identify neurons that exhibit stable tuning over several days. In contrast,

we can't determine whether some neurons have unstable tuning over days since, even if we recorded such neurons during multiple days, we wouldn't be able to determine that the recordings originate from the same neuron. Thus, this figure demonstrates that some neurons can maintain a stable 3D tuning across days but doesn't imply that the 3D tuning of some HD cells can't drift over days. Over 549 cells, we recorded Experiment 3-L on 2 distinct days in 63 cells, 3 days in 15 cells, and 4 days or more in 13 cells.

(c) 3D tuning curve of the example cell (conjunctive cell in CIN; same as in **Supplementary Movie 8**). A vertical section of the 3D tuning curve is shown at an azimuth of 0° that corresponds to the cell's PD. Data averaged across all repetitions of Experiment 3-L.

(d) Reproducibility of the tuning curve. The cell's response was recorded 4 times during Experiment 3-L, on 4 distinct days spanning a 2-week period. 3D tuning curves were recomputed for each repetition. The same vertical section as in (a) is shown for all repetitions (labelled 1 to 4), using the same color scale. Peak firing occurs consistently in the vicinity of ND orientation.

(e) We evaluated tuning stability by fitting the 3D model to the 4 tuning curves, and computing the pixel-by-pixel correlation between the model fits and the raw curves. The correlations are shown on a matrix; the average correlation over off-diagonal elements, i.e. across different repetitions, is 0.67.

(f) Azimuth tuning curve in upright orientation extracted from the 4 3D tuning curves, and centered on each curve's PD.

(g) Pitch tuning curve extracted from the 3D tuning curves, at the azimuth corresponding to each curve's PD. Analyses in panels d to g indicate that 3D responses (or responses along 1D yaw and pitch trajectories) were stable across several days in the example cell.

(h) Distribution of the average correlation between repetitions of Experiment 3-L (as in panel e), for all tuned cells (n= 90 cells; 47 conjunctive, 40 tilt only, 3 azimuth-only). We used a shuffling procedure to determine the threshold value over which the correlation is significant (at $p<0.01$) on a cell by cell basis. The average threshold across cells is 0.26 (±0.04 s.d., interval shown in grey). 94% of cells pass the significance threshold, with the median correlation being 0.63 ([0.57-0.67] CI), similar for conjunctive and tilt-only cells (Wilcoxon rank sum test, $p=0.46$) and cells with PD in the pitch and roll planes ($p=0.49$).

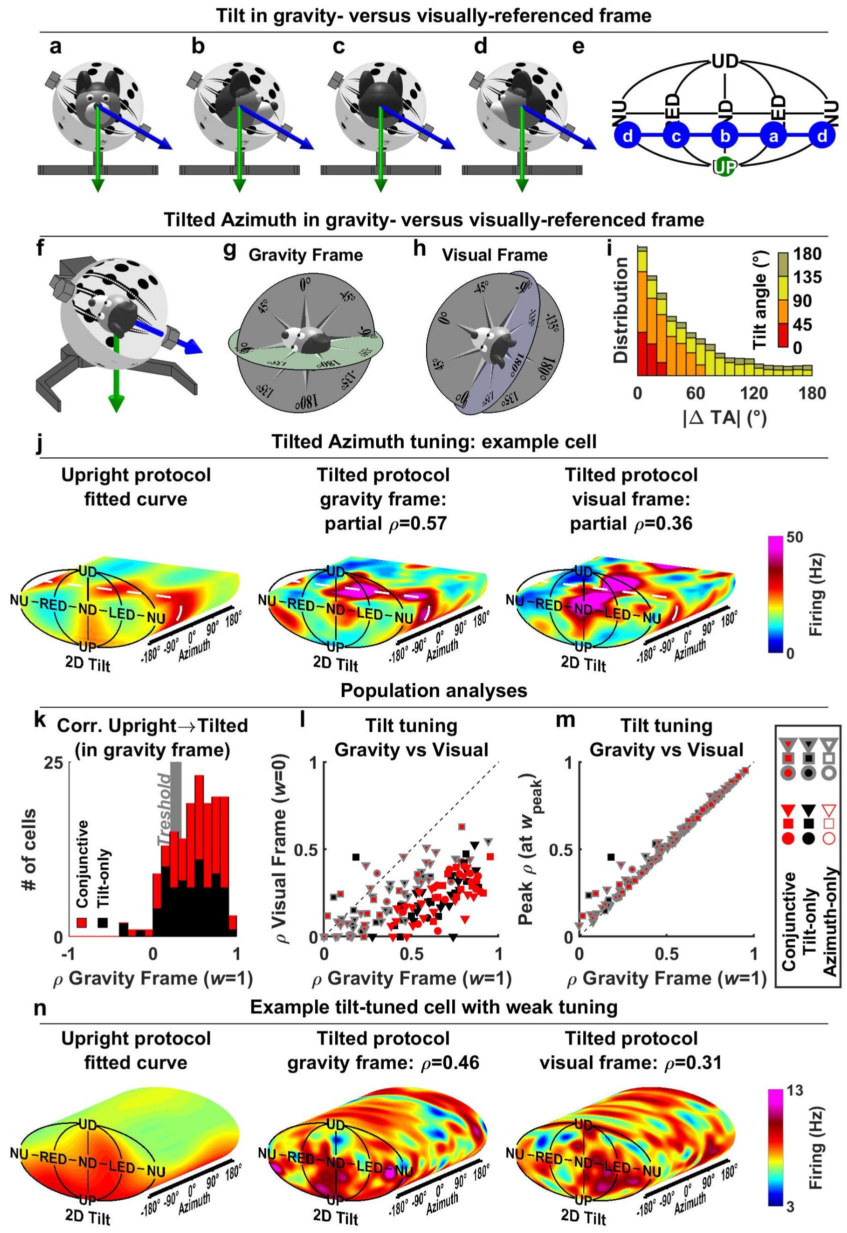

Supplementary Figure 15: Protocol 3-T.

(a-e) Comparison of tilt tuning computed relative to gravity-referenced (green) or visually-referenced (blue) vertical. Panels a-d illustrate position situations where the head is upright relative to gravity (UP point in panel e) but tilted 60° (along4 different directions) relative to the visually-referenced vertical axis (marked a-d in panel e). As a rule, during the 3D rotation protocol, the gravity-referenced and visually-referenced verticals always differ by exactly 60°.

(f-i) Comparison of TA azimuth measured in a gravity- or visually-referenced frame. Panel f: example 3D head orientation. Panels g-h: representation of the earth-horizontal compass in a

gravity-referenced (f, green) and visually-referenced (h, blue) frame. Tilted azimuth is measured by rotating the earth-horizontal compass in alignment with the head-horizontal plane, resulting in the grey compasses in panels g-h. In this example, the resulting azimuth is 60° and 2° in panels g and h respectively, i.e. a difference of 58°. Panel i: the difference between TA in a gravity-referenced or visually-referenced frame is a complex function of 3D head orientation. To appreciate how they differ in practice, we computed the difference in TA between these frames (|Δ TA|) over the whole protocol, color-coded as a function of tilt angle (in either frame). Although the |Δ TA| is minimal for small head tilts (e.g. <45°, red: median |Δ TA| = 10°), it increases for larger tilt angles (e.g. larger than 45° and lower 135°; orange-yellow; median |Δ TA|=37°). Since HD cells still exhibit appreciable azimuth responses in this range of tilt, it is possible to use this protocol to determine the frame in which they encode TA.

(j) Example conjunctive cell. Left: reference tuning function fitted to the response measured with the rotator upright. A section of the tuning curve at 105° tilt is shown. The cell fires preferentially at an azimuth of -77° (broken white line). Middle and right: experimental tuning curves measured with the rotator tilted and computed with tilt expressed in a gravity-referenced frame, and azimuth expressed in a gravity-referenced (middle) or visually-referenced (right) frame. Azimuth tuning is well preserved when expressed in a gravity-reference frame (middle) but not in a visually-referenced frame (left). Accordingly, the partial correlation between the reference tuning curve (left) and the experimental tuning curves (middle, right) is higher when azimuth is expressed in a gravity-reference frame (ρ=0.32 versus 0.13; partial correlation computed by removing the effect of gravity tuning, see Methods).

(k) Stability of 3D tuning when recorded with the rotator upright and tilted. We plot the distribution of ρ computed in a gravity-referenced frame (see **Fig. 6a,b**). We used a permutation test to determine if ρ was significantly higher than 0 on a cell-by-cell basis (mean threshold value: 0.11; SD=0.03; vertical grey band). ρ was significantly higher than 0 in 135/148 tilt-tuned cells (19/22 ADN; 38/46 RSC; 68/80 CIN; permutation test, p<0.01). Cells in which ρ was not significantly higher than 0 typically have lower peak firing rate (5.7 vs 13.2 Hz, p=0.007, Wilcoxon rank sum test). The median value of ρ was 0.53 ([0.45-0.57] CI).

(l,m) Additional analyses of tilt tuning (see **Fig. 6**). Panel l: Even though, at the population level, w_{peak} accurately centered on 1 (**Fig. 6d**), we observed that it was close to zero (or at least lower than 0.5) in a few cells (3%). To investigate whether a subpopulation of cells may encode visually-referenced tilt, we tested if the correlation ρ is significantly higher when tilt is expressed in a gravity or visual frame on a cell-by-cell basis. As expected, ρ was significantly higher in a gravity reference frame in 78/148 cells (15/22 ADN; 26/46 RSC; 37/80 CIN). The difference between the two frames was non-significant in all other cells (markers with grey border) but one black (marker about the diagonal), which is weakly modulated cell and likely a false positive. Panel m: we test if some cells may use an intermediate reference frame by comparing the correlation when tilt is expressed in a gravity reference frame (w=1) or at the peak of the distribution (w_{peak}). Data points lie close to the diagonal since the peak is generally close to 1. Importantly, the peak correlation is significantly higher than the correlation in a gravity frame in only one cell (likely false positive, same as in panel k), indicating that cells don't use intermediate reference frames.

(n) Example tilt-tuned cell where the correlation ρ was similar in a gravity-referenced (middle panel) and visually- referenced (right) frame. This cell had a low modulation amplitude (tilt modulation amplitude = 2.9 Hz; NTA=0.32). As a result, the tuning curves recorded with the rotator tilted where markedly flat and noisy in both reference frames; and the correlations weren't significantly different. Similarly, cells where the correlations in gravity- and visually referenced frames (symbols with grey outlines in panel l) had lower tilt modulation amplitude (median peak-valley modulation: 4.2 versus 6.35 Hz, p=0.002, Wilcoxon rank sum test; median NTA = 0.39 versus 0.52, p=0.002) and, being comparatively noisier, were generally poorly correlated (median ρ: 0.3 versus 0.67 in gravity frame, 0.18 versus 0.24 in visual frame).

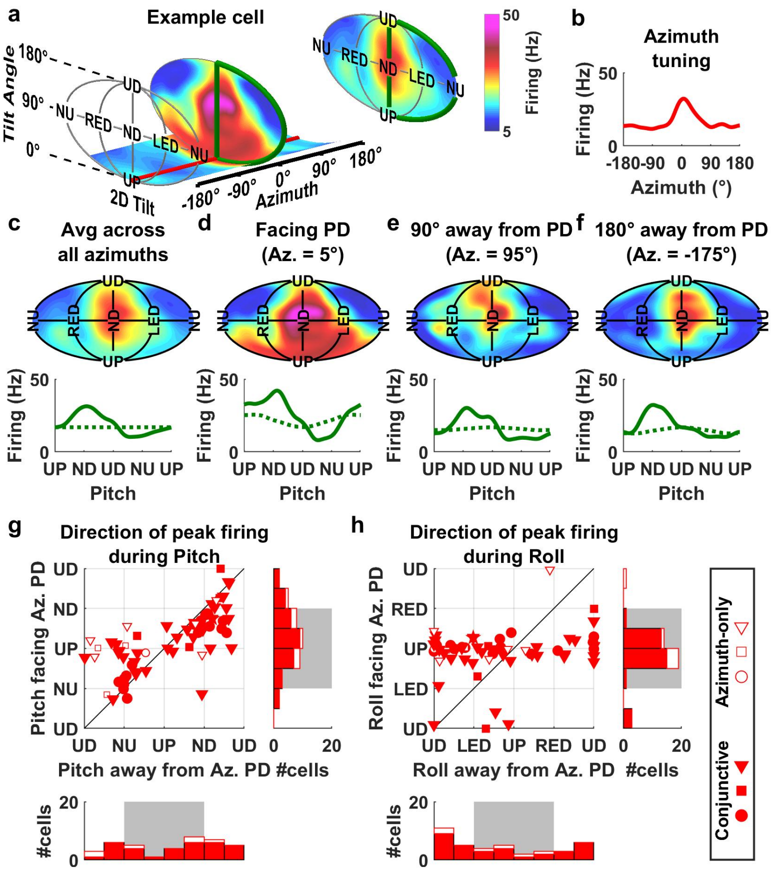

Supplementary Figure 16: Possible bias when measuring pitch/roll tuning in azimuth-tuned cells (see also Laurens and Angelaki, 2019, ref[10]). A recent study in rat ADN (Shinder and Taube, 2019, ref[11]) failed to identify tilt responses in a sample of 24 azimuth-tuned HD cells. In that study, mice were positioned upright, facing the azimuth PD, and rotated in pitch and/or roll. The authors observed that most cells fired more in tilt positions near upright and concluded on that account that there is “limited evidence that cells contained conjunctive firing with pitch or roll position” (sic). Here we demonstrate that the experimental protocol used by Shinder and Taube[11], where mice were tilted while facing the cell’s PD, tends to conceal pitch/roll tuning, because it is superimposed on a strong azimuth tuning, whose strength is reduced as a function of head tilt (**Fig. 3f,g**). We show that, had we used the same experimental protocol and analyses, we would have failed to see robust tilt tuning as well.

(a) 3D tuning curve of an example conjunctive cell (measured during Experiment 3-L; same cell as in **Fig. 4c** and **Supplementary Fig. 14**). When averaged across all azimuths (rightmost plane), the cell is tuned to tilt with a PD in ND. The cell is also tuned to azimuth, with a PD at 5°.

A vertical section (i.e. firing rate for all tilt positions at a given azimuth) of the tuning curve is shown at the azimuth PD. When exclusively tested during pitch in this plane (green line; as Shinder and Taube, 2019[11], did), the cell's tilt modulation is much broader and the cell's firing at ND is barely above its firing when the animal is upright (red).

(b) Average azimuth tuning curve when upright, peaking at 5°.

(c) Analysis in this study: Upper panel: Tilt tuning curves averaged across all azimuth angles (as in panel a). Lower panel: Firing rate measured along a pitch trajectory; Solid green curves: actual data; Dashed green curves: simulated data (see below).

(d) Experiment by Shinder and Taube, 2019[11]: Tilt tuning was tested when animals faced the azimuth PD. Note that firing is elevated in the vicinity of upright. Even though the firing is largest in ND, the preferred pitch direction, evaluated by fitting a von Mises function, is biased towards upright (67° ND tilt in d versus 108° ND tilt in c).

(**e,f**) Tilt tuning tested 90° (e) or 180° (f) away from the cells' azimuth PD. Firing measured during pitch rotation away from the PD is similar as the average curve in (c). We use the 3D model fit to demonstrate that the curve in (d) is affected by azimuth tuning. We fit the cell's 3D tuning curve, then alter the model's parameter to eliminate tilt tuning (by setting A to 0 and FR_0 to the cell's average firing in $FR_{Ti}(\alpha,\gamma)$; see **Methods**). Next we simulate the pitch tuning curve (dashed green curves) that is now influenced entirely by its azimuth tuning. The resulting curve peaks in UP orientation in (d), but is flat in other panels. This indicates that azimuth tuning affects the cell's response when facing the azimuth PD (d), such that it biases the firing rate towards upright (by interacting multiplicatively) but has little effect when facing away from the azimuth PD (e,f) or when data are averaged across all azimuths (c). We note that the green curve in (d) resembles most example pitch or roll tuning curves shown by Shinder and Taube's study[11]. Based on these simulations, we predict that, had the authors analyzed individual pitch/roll tuning curves recorded when the mouse faced away from the cell's PD (e, f), they would have seen tilt tuning with preferred tilt away from upright.

(g,h) Same analysis, at the population level. We simulated pitch/roll rotations for all azimuth-tuned cells that were also azimuth tuned in the rotator (n=63; 53 conjunctive and 10 azimuth-only cells). Top: Scatter plot showing how pitch rotations while facing the cell's azimuth

PD can bias conclusions. Peak responses (by fitting von Mises functions) to pitch and roll rotations when facing the azimuth PD (ordinate; as in Shinder and Taube's study[11]) and when facing away from the azimuth PD (abscissa). Bottom and right: Marginal distributions are shown as histograms.

When pitching while facing the azimuth PD (panel g), most conjunctive cells fire preferentially close to upright right-side histogram, grey zone, red bars (41/53, 78%, $p<10^{-4}$), similar to the example cell in a-f. When adding azimuth-only cells (open symbols/bars), the proportion of cells firing preferentially close to upright is maintained at 49/63 (77%). The bias is even more drastic in roll (panel h; because tilt tuning is weaker in roll), with the 49/53 conjunctive cells firing preferentially around upright (lower histogram, grey zone, red). In contrast, when pitching or rolling away from the azimuth PD (g,h; abscissae), half of the conjunctive cells (solid red symbols/bars, 24/53 in g, 19/53 in not significantly different from 50%, p=0.6/0.05 respectively) fire preferentially close to upright (grey band in the marginal distribution) and the other half fire preferentially closer to UD. Thus, while recording from our neurons, if we had done the experiment (pitch/roll when animal faced azimuth PD in a small sample of cells) and analyses as in Shinder and Taube's study[11], we would likely not have been able to identify tilt tuning.

We conclude that our dataset and quantitative analyses predict that, even though the PD of tilt tuning is distributed uniformly between upright and inverted orientation (**Fig. 5a**), conjunctive cells would appear to respond preferentially in upright orientation when recorded and analyzed as in Shinder and Taube's study[11]. The results published in our study are therefore entirely compatible with those described by Shinder and Taube's study[11]. The conclusions are opposite because the systematic scanning of 3D orientation (rather than a limited subset) allowed us to reveal tilt tuning, that was concealed by azimuth tuning in the Shinder and Taube's study[11]. For more details about modeling the experimental findings of Shinder and Taube[11], see Laurens and Angelaki (2019)[10].

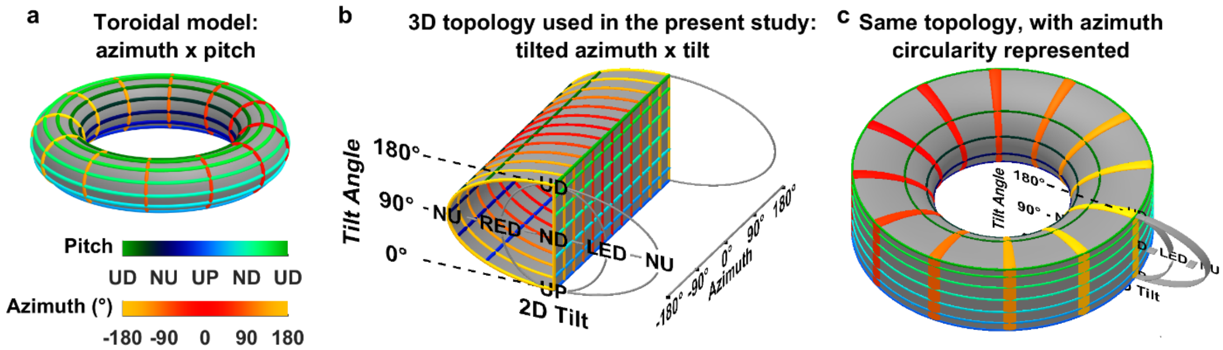

Supplementary Figure 17: Comparison between the separable, multiplicative model of Fig. 4 and toroid topology[9].

(a) The toroid model is restricted to tilt movements in pitch, and assumes that azimuth and pitch are independent, i.e. pitch movements don't change azimuth. Combination of pitch and azimuth can be represented on the surface of a torus. Iso-pitch lines (green/blue/black color code), that correspond to one pitch orientation and all possible azimuths, form horizontal circles. Iso-azimuth lines (yellow/red color scale), that correspond to one azimuth angle and all possible pitch tilts form vertical lines.

(b) Representation of the same iso-pitch and iso-azimuth lines in the 3D topology used in this study, when azimuth is expressed in a TA frame. Each iso-azimuth line forms a D-shaped curve that passes through UP, NU, UD and ND orientation, and each iso-pitch line forms a horizontal line.

(c) When the diagram in (b) is looped upon itself to account for the circularity of azimuth (note that this representation was not used outside of this figure because it distorts volumes), the surface formed by iso-azimuth and iso-pitch lines adopts a toroidal topology identical to (a). Thus, the 3D model used here is equivalent to the toroidal topology in (Finkelstein) if (1) tilt is restricted to the pitch plane and (2) azimuth is expressed in the TA frame.

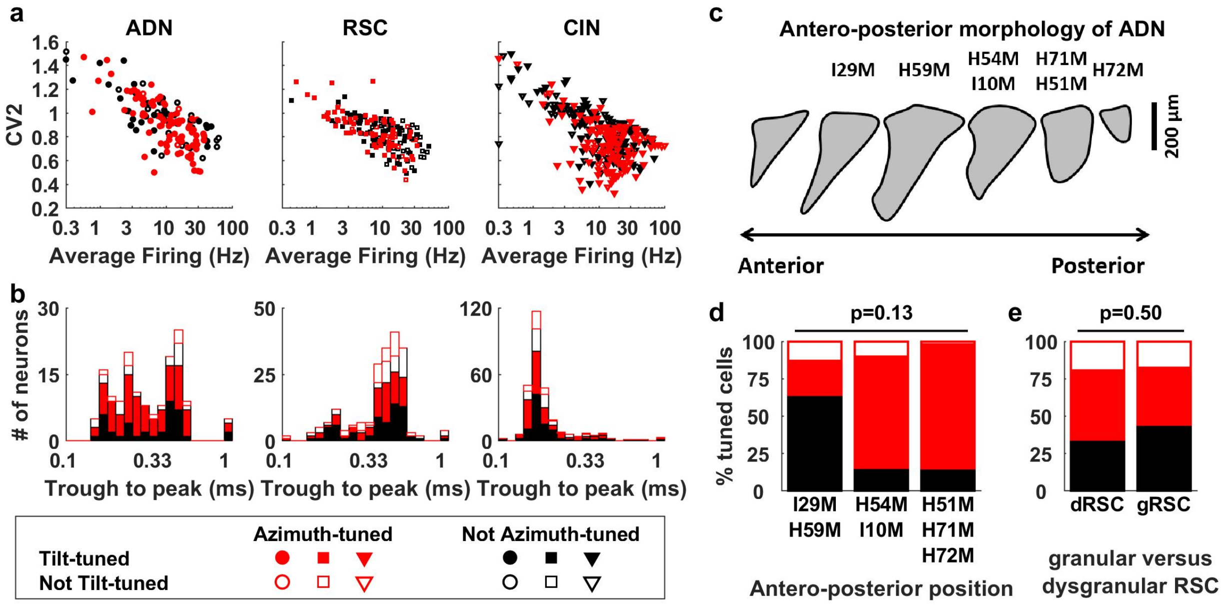

Supplementary Figure 18: Firing properties and spatial distribution of responses. To better assess population responses in ADN, we included 3 additional animals (H51M, H54M and H59M) in which cells could be classified using the same criteria as in other animals.

(a) Scatter plot of CV2 vs. average firing rate during freely moving in the arena. Median firing rate: 10 Hz in ADN, 11 Hz in RSC, 13 Hz in CIN. Median CV2: 0.93 in ADN, 0.84 in RSC, 0.79 in CIN. CV2 varied significantly across areas (Kruskal-Wallis non-parametric ANOVA, $p = <10^{-8}$), but firing rate was similar (p=0.06).

(b) Trough to peak duration of action potentials. Most (91%) cells in RSC have long trough to peak spike duration (>0.33ms), whereas most (74%) cells in CIN have short spike duration. About half (53%) of cells in the ADN have short spike duration; the proportions of conjunctive, tilt-only, azimuth-only and non-responsive cells are 23%, 59%, 12% and 6% respectively amongst ADN neurons with short spike duration and 30%, 49%, 14% and 7% amongst ADN neurons with long spike duration: these proportions are similar across neurons with short- and long-duration spikes (Chi square test, p = 0.77, χ^2=1.1, 3 dof). The CIN is a fiber bundle and neuronal activity recorded therein is therefore expected to consist of axonal spikes, that can be recorded by tetrodes[12] and typically exhibit small duration[13]. Note that most units recorded in the ADN also had short-duration spikes. See Laurens et al (2019)[14] for further analyses of spiking activity in these areas.

(c,d) Distribution of tilt- and azimuth-tuned cells along the antero-posterior axis of the ADN. (c) We identified the position of tetrode tracks in all animals by examining the cross-section of the ADN along successive brain sections. The anterior portion of the ADN has a triangular shape, which elongates into a narrow triangle before reaching a maximal cross-section size. Further along the posterior axis, the ADN decreases in size and adopts a rounder shape. The position of each animal is indicated. (d) Percentage of conjunctive, tilt-only and azimuth-only cells. The animals are pooled in 3 groups based on recording position corresponding to animals where recordings were at the most anterior, intermediate or most posterior position. The proportion of azimuth-tuned cells is lower in animals I29M and H59M, where tetrodes were placed in the anterior ADN over those recorded in intermediate and posterior AND (open/solid red bars, 36% versus 84%). However, the number of recorded animals is too low to establish whether the distribution of cell type depends significantly on recording position (p=0.13, Chi-square statistic, statistical significance evaluated by shuffling the recording position along animals).

(e) Percentage of conjunctive, tilt-only and azimuth-only cells in granular versus dysgranular RSC. We found no difference in the proportions of cells between these areas.

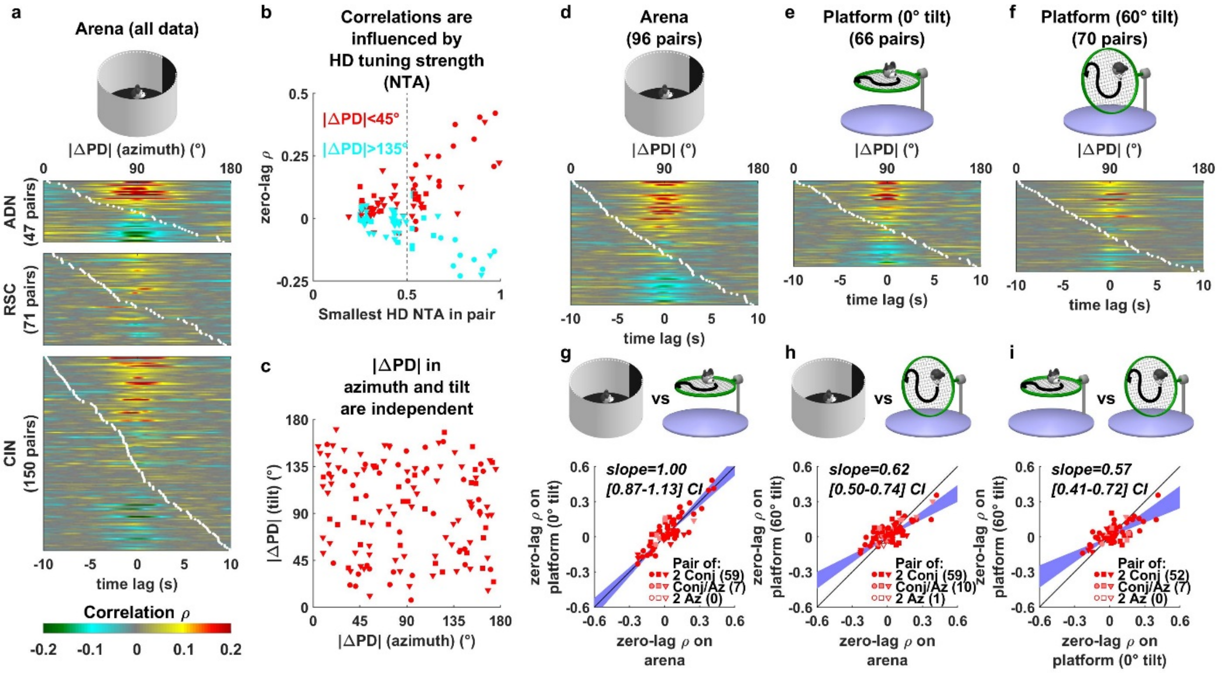

Supplementary Fig. 19: The stability of the 1D attractor weakens when the head tilts. When the head is upright, the HD system is classically described as a 1D attractor where azimuth-tuned cells with similar PD tend to fire together. Our findings question whether this attractor persists during 3D motion since (1) conjunctive cells with similar preferred azimuth direction but different preferred tilt would fire at different tilt orientations, and (2) the tuning of azimuth-tuned cells weaken when the head tilts. This suggests that the 1D attractor may weaken or disappear when tilted. We tested this hypothesis by following the analysis in ref[4]. This study was based on the cross-correlation between simultaneously recorded cells: HD cells with similar PD tend to fire together whereas HD cells with opposite PD tend to be anti-correlated.

(a) Firing cross-correlograms (correlation as a function of time lag, lower abscissa) of all pairs of azimuth-tuned cells recorded simultaneously in the arena. Within each brain region, cell pairs are ordered based on the distance between their azimuth PD (|ΔPD|, white, upper abscissa). As expected, cells with similar PD (upper portion of the graphs) tend to be fire simultaneously (positive correlation at zero time lag, yellow/red colors) whereas cells with opposite PD (lower parts of the graphs) rarely fire simultaneously (negative correlation, cyan/green colors).

(b) Correlation at zero time lag, expressed as a function of the lowest HD tuning strength of both cells in the pair. As expected, pairs with similar (red) or opposite (cyan) PD are positively/negatively correlated when both cells exhibit strong HD tuning (e.g. NTA>0.5). In contrast, these correlations are close to zero when at least one cell in the pair is weakly tuned (NTA<0.5). In the following analyses, we exclude cells pairs where the lowest NTA is less than 0.5 from panels (d-i) (although statistical analyses with all pairs included are mentioned in legend of panels g-i).

(c) Before proceeding further, we verify that cells with similar/opposite azimuth PD don't have similar/opposite tilt PD. Data shown is from 140/240 (58%) pairs of conjunctive cells where tilt tuning was recorded in the rotator. We find that differences are independent one from another (Spearman correlation rank = -0.01, p=0.6).

(d-f) We now select pair of cells with strong HD tuning (NTA>0.5, see panel b) and compare the correlations between cell pairs in the arena (d) and when walking on the platform in 3D. As a control, we separate recordings performed when the platform is horizontal (e) and when it is tilted by 60° (f). Note that we included all cells recorded on the platform, even if they didn't pass the criterion for uniform coverage of 3D space (see **Methods**, section Neuron selection and inclusion criteria, and **Table 1**), which is not important in the present analysis. Cells from all brain regions are pooled. As in (a), cells with similar/opposite PD exhibited positive/negative correlations at zero lag on all setups. Yet, further analysis revealed qualitative differences between these conditions.

(g-i) Comparison of the zero-lag correlations between conditions. Pairs are color-coded to indicate whether both cells are conjunctive (solid red), azimuth-only (open markers) or whether the pair contains one conjunctive and one azimuth-only cell (pink markers): note that most pairs are made of conjunctive cells. Zero-lag correlations were indistinguishable in the arena or on a horizontal platform (panel g; type II regression, slope not different from 1, bootstrap analysis). The slopes are similar if the inclusion criterion in panel b is lifted (0.97 [0.84 - 1.09] CI, 0.64 [0.5 - 0.77] CI and 0.58 [0.45 - 0.73] CI in panels g, h and I respectively). In contrast, zero-lag were significantly reduced when the platform was tilted, compared to when exploring the arena

(h) or a horizontal platform (i) (bootstrap analysis, the confidence interval of the slopes don’t overlap the confidence interval in (g)).

Conclusion: According to the neural attractor theory, azimuth-tuned cells with similar PD activate each other through reciprocal connections, which causes them to discharge together, whereas cells with opposite PD inhibit each other. This forms a 1D neural attractor where the population responses is always a packet of nearby active cells. Yet, most HD cells are in fact composite (g-i); thus, HD cells with similar preferred azimuth may have distinct preferred tilt (c). When the head tilts, we expect that such cells cease firing together, and this hypothesis is supported by the present analysis. We conclude that the HD system follows a 1D attractor dynamics when the head is upright, but not when the head tilts.

	Animal name	*H51M*	*H54M*	*H59M*	H71M	H72M	I10M3	I29M	H65M	H68M	H69M	H74M	AA1	AA2	AA18	AA20	Total
	Region	*ADN*	*ADN*	*ADN*	ADN	ADN	ADN	ADN	CIN	CIN	CIN	CIN	RSC	RSC	RSC	RSC	
	**Total**	***14***	***37***	***42***	**35**	**13**	**6**	**15**	**72**	**137**	**61**	**27**	**53**	**124**	**8**	**29**	**580**
	Azimuth tuned	*6*	*30*	*12*	33	9	6	4	34	68	29	17	26	58	0	20	304
	not Azimuth tuned	*8*	*7*	*30*	2	4	0	11	38	69	32	10	27	66	8	9	276
**Experiment 2**	**Platform**	***0***	***0***	***0***	**19**	**0**	**5**	**5**	**0**	**19**	**14**	**9**	**28**	**40**	**0**	**0**	**139**
	Conjunctive (Az&Tilt)	*0*	*0*	*0*	16	0	5	3	0	5	5	3	5	14	0	0	56
	Tilt-only	*0*	*0*	*0*	0	0	0	1	0	6	3	4	6	16	0	0	36
	Azimuth-only	*0*	*0*	*0*	1	0	0	0	0	1	2	1	9	5	0	0	19
	Not HD	*0*	*0*	*0*	2	0	0	1	0	7	4	1	8	5	0	0	28
	Additional cells in Fig. S20	*0*	*0*	*0*	*1*	*0*	*1*	*0*	*0*	*20*	*7*	*7*	*0*	*19*	*0*	*0*	*55*
**Experiment 3L,D,T**	**Rotator - in Light**	***14***	***37***	***40***	**30**	**13**	**3**	**14**	**72**	**137**	**59**	**19**	**53**	**114**	**8**	**27**	**549**
	Conjunctive (Az&Tilt)	*6*	*27*	*6*	30	8	2	3	28	47	25	11	14	37	0	17	222
	Tilt-only	*5*	*6*	*18*	0	3	0	7	23	45	19	3	15	43	1	7	166
	Azimuth-only	*0*	*3*	*5*	0	1	1	0	6	21	3	2	12	17	0	1	64
	Not HD	*3*	*1*	*11*	0	1	0	4	15	24	12	3	12	17	7	2	97
	**Rotator - in Darkness**	*0 (0)*	*0 (0)*	*26 (13)*	12 (12)	5 (4)	2 (1)	8 (6)	47 (33)	98 (68)	43 (32)	0 (0)	0 (0)	44 (30)	5 (1)	26 (23)	290 (210)
	**Rotator - Tilted**	*0 (0)*	*0 (0)*	*24 (12)*	12 (12)	5 (3)	1 (1)	8 (6)	41 (26)	55 (38)	22 (16)	0 (0)	0 (0)	38 (27)	6 (1)	20 (18)	208 (148)
**Experiment 4: yaw, pitch, roll**		*0 (0)*	*0 (0)*	*0 (0)*	0 (0)	0 (0)	0 (0)	0 (0)	40 (28)	18 (14)	11 (8)	0 (0)	0 (0)	0 (0)	0 (0)	0 (0)	69 (50)

Supplementary Table 1: Number of recorded cells and categories. For each mouse (and each area), the table indicates the number of cells recorded during Experiment 1 (numbers in top row), 2, 3 and 4), along with their categorization. The last column shows the total number of neurons tested for each experimental protocol. Grey lines and column: animals and cells that didn't pass the general inclusion criteria and are included only in specific Supplementary analyses. Animals H51M, H54M and H59M were recorded in an earlier version of the rotator (without orienting stripes) and are used only in Supplementary Fig. 18. These animals are not counted in the total number of cells. Some cells recorded on the platform were excluded as the animals didn't cover 3D space well enough to compute 3D tuning curves, but were included in **Supplementary Fig. 19**.

Experiment name	Illustration	Description	Goal
Experiment 1-L0	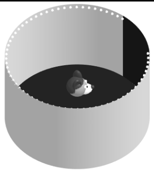	Free motion in arena.	Measure azimuth tuning using traditional method.<br>Control for tuning stability (by comparing with Experiment 1-L1,2).
Experiment 2	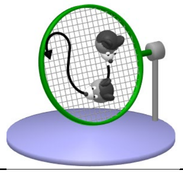	Free motion on orientable platform.	Measure 3D tuning in freely moving animals at up to 60° tilt.
Experiment 3-L	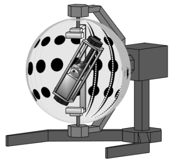	3D tuning curve scanning in light.	Measure tuning uniformly in entire 3D space.
Experiment 3-D	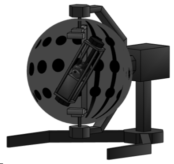	3D tuning curve scanning in darkness.	Test that tilt tuning depends of gravity and not visual cues.
Experiment 3-T	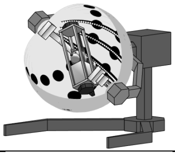	3D tuning curve scanning in a tilted visual surround.	Test that tilt tuning depends of gravity and not visual cues.
Experiment 4	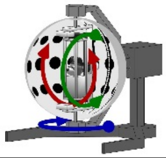	Rotations in yaw, pitch and roll (in light and darkness)	Test that 3D tuning is conserved during simple trajectories.
Experiment 1-L1	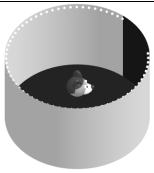	Free motion in arena.	Measure azimuth tuning using traditional method.<br>Control for tuning stability (by comparing with Experiment 1-L0,2).
Experiment 1-D	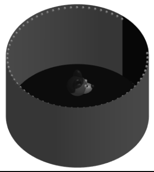	Free motion in arena, in darkness.	Test that azimuth tuning is maintained in darkness.
Experiment 1-L2	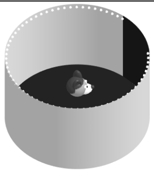	Free motion in arena.	Measure azimuth tuning using traditional method.<br>Control for tuning stability (by comparing with Experiment 1-L0,1).

Supplementary Table 2: Description and order of experimental protocols.

Supplementary References

1. Lein, E. S. *et al.* Genome-wide atlas of gene expression in the adult mouse brain. *Nature* **445**, 168–176 (2007).
2. Franklin, K. B. & Paxinos, G. *The mouse brain in stereotaxic coordinates*. vol. 3 (Academic press, New York, 2008).
3. Taube, J. S. The Head Direction Signal: Origins and Sensory-Motor Integration. *Annu. Rev. Neurosci.* **30**, 181–207 (2007).
4. Peyrache, A., Lacroix, M. M., Petersen, P. C. & Buzsáki, G. Internally organized mechanisms of the head direction sense. *Nat. Neurosci.* **18**, 569–575 (2015).
5. Yoder, R. M. & Taube, J. S. Head Direction Cell Activity in Mice: Robust Directional Signal Depends on Intact Otolith Organs. *J. Neurosci.* **29**, 1061–1076 (2009).
6. Park, E. H., Keeley, S., Savin, C., Ranck, J. B. & Fenton, A. A. How the Internally Organized Direction Sense Is Used to Navigate. *Neuron* **101**, 285-293.e5 (2019).
7. Jacob, P.-Y. *et al.* An independent, landmark-dominated head-direction signal in dysgranular retrosplenial cortex. *Nat. Neurosci.* **20**, 173–175 (2017).
8. Laurens, J. & Angelaki, D. E. The Brain Compass: A Perspective on How Self-Motion Updates the Head Direction Cell Attractor. *Neuron* **97**, 275–289 (2018).
9. Finkelstein, A. *et al.* Three-dimensional head-direction coding in the bat brain. *Nature* **517**, 159–164 (2015).
10. Laurens, J. & Angelaki, D. E. A model-based reassessment of the three-dimensional tuning of head direction cells in rats. *J. Neurophysiol.* **122**, 1274–1287 (2019).
11. Shinder, M. E. & Taube, J. S. Three-dimensional tuning of head direction cells in rats. *J. Neurophysiol.* **121**, 4–37 (2019).
12. Robbins, A. A., Fox, S. E., Holmes, G. L., Scott, R. C. & Barry, J. M. Short duration waveforms recorded extracellularly from freely moving rats are representative of axonal activity. *Front. Neural Circuits* **7**, (2013).
13. Barry, J. M. Axonal activity in vivo: technical considerations and implications for the exploration of neural circuits in freely moving animals. *Front. Neurosci.* **9**, 153 (2015).

14. Laurens, J. *et al.* Multiplexed code of navigation variables in anterior limbic areas. *bioRxiv* 684464 (2019) doi:10.1101/684464.
