# Supplementary material for: A gravity-based three-dimensional compass in the mouse brain
Source: Nat Commun. 2020 Apr 15;11:1855. doi: 10.1038/s41467-020-15566-5 (PMC7160108; doi:10.1038/s41467-020-15566-5)
Supplement: Supplementary file 4 — Description of Additional Supplementary Files [file 41467_2020_15566_MOESM4_ESM.pdf]

Description of Additional Supplementary Files

File Name: Supplementary Movie 1
Description: Volumetric representation of 3D head orientation. Head tilt relative to vertical is represented on a plane (labelled 2D Tilt; see Supplementary Fig. 5) and head azimuth is represented on a third (unfolded) axis to form a 3D volume. The animation represents a mouse head placed at 5 different tilt orientations (upright, 60° LED, 90° ND and NU, 150° LED) and moved though all possible azimuths. The center of the head is placed at the corresponding 3D position in the volumetric plot. The movements that change azimuth without changing head tilt, correspond to rotations around an earth-vertical axis, as represented by red arrows.

File Name: Supplementary Movie 2
Description: 3D tuning curve of the example neuron in Fig. 1g. The tuning curve of Fig. 1g is shown as animation sweeping through tilt angles from 0 to 60°. The lower right panel shows the average azimuth tuning curve at each tilt angle, computed by averaging across all tilt orientations. The cell is tuned to tilt, with a preferred orientation at 48° ND, but not to azimuth.

File Name: Supplementary Movie 3
Description: Three-dimensional passive re-orientation protocol in rotator (Fig. 1h; see also Supplementary Fig. 6) and response of a tilt-tuned RSC cell. Upper left panel: an animated model of the 3D rotator (the rotator is ~2.4 m height; the size of the mouse head is exaggerated) displays its position recorded in real-time using potentiometers during Experiment 3-L. Lower panel: 3D head orientation is shown in volumetric space. Instantaneous head orientation is represented by a black circle; the travelled trajectory by a grey line, and recorded spikes by red dots. The trajectory is also projected on a tilt plane (right panel). A clock shows the time elapsed since the beginning of the recording. The first 10s of recording are shown in real time and then the movie accelerates to span the entire experiment. Spikes clearly cluster in a space that corresponds to ND tilt between 120 and 150°, independently of azimuth. The end of the movie (starting at t=50s) shows the complete tuning curve as a color map. The animation sweeps through all tilt angles from 0 to 180° to allow visualizing the entire 3D volume. The cell is tuned to tilt, with a preferred orientation at 135° ND, but not to azimuth.

File Name: Supplementary Movie 4
Description: 3D tuning curve of the example neuron in Fig. 1i (conjunctive cell). The movie pauses at a tilt angle of 10° to show azimuth tuning, which is strongest in the vicinity of upright. The cell is also tuned to tilt, with a peak response in NU orientation. Same format as in Supplementary Movie 2.

File Name: Supplementary Movie 5
Description: 3D tuning curve of the example neuron in Fig. 1j (tilt-only cell). Same format as in Supplementary Movie 2.

File Name: Supplementary Movie 6

Description: Azimuth tuning of an example neuron in YO, EH and TA frames. Upper left: animated model of the rotator during Experiment 3-L. Right panels: head tilt (α) versus azimuth computed in a yaw-only frame (upper panel), earthhorizontal frame (middle) or tilted azimuth frame (bottom). The tuning curves of an example conjunctive ADN cell, obtained by averaging the 3D tuning curve across all tilt orientations (γ), are shown as a color map. As the animation runs, the position of the head and the spikes emitted by the cells are indicated in each panel. The position is identical in the 3 frames during the first few seconds of the experiment, because the head is about upright where all frames are identical. As soon as the head tilts beyond 90°, the 3 frames diverge. When the head comes back to upright orientation, the position is again identical in the EH and TA frame because these frames maintain allocentric invariance. In contrast, azimuth in the yaw-only frame can't track allocentric head position (as shown in Fig. 3c). As shown by the spiking activity and the tuning curves, the cell is tuned to azimuth in the EH and TA frame. Beyond 90° tilt, where the EH and TA frame diverge most, azimuth tuning is lost in the EH frame but maintained, although weakened, in the TA frame. The final sequence of the Supplementary Movie shows the cell's full 3D tuning curve. Same format as in Supplementary Movie 2.

File Name: Supplementary Movie 7
Description: 3D tuning curve of the example azimuth-only neuron in Fig. 3e. The movie pauses at tilt angles of 30° and 110°. As shown by the azimuth tuning curve (lower right panel), azimuth tuning fades away when tilt angle increases. Same format as in Supplementary Movie 2.

File Name: Supplementary Movie 8
Description: 3D tuning curve of the example conjunctive neuron in Fig. 4e. The neuron is tuned to tilt with a preferred orientation at 42° ND, and to azimuth with a PD of -27°. Consequently, the cell fires maximally at 3D orientations centered on these coordinates. Same format as in Supplementary Movie 2.

File Name: Supplementary Movie 9
Description: 3D tuning curve of the example conjunctive neuron in Fig. 4f; Supplementary Fig. 14. The neuron is tuned to tilt with a preferred orientation at 105° ND, and to azimuth with a PD of 5°. Although azimuth tuning is clear in the lowest portion of the tuning curve, it decreases with tilt angle and is therefore minimal at 105°, where the cell appears to be only tuned to tilt. As a consequence, the cell seems to alternate from azimuth tuning when the head is close to upright to tilt tuning at large tilt angles. Same format as in Supplementary Movie 2.
